# Evaluating antimicrobial resistance in the global shrimp industry

**DOI:** 10.1111/raq.12367

**Published:** 2019-07-08

**Authors:** Kelly Thornber, David Verner‐Jeffreys, Steve Hinchliffe, Muhammad Meezanur Rahman, David Bass, Charles R. Tyler

**Affiliations:** ^1^ Centre for Sustainable Aquaculture Futures University of Exeter Exeter UK; ^2^ Biosciences University of Exeter Exeter UK; ^3^ Centre for Environment, Fisheries and Aquaculture Science Weymouth UK; ^4^ Department of Geography University of Exeter Exeter UK; ^5^ Worldfish Bangladesh World Fish Bangladesh Office Dhaka Bangladesh

**Keywords:** antibiotics, antimicrobial resistance, aquaculture, international trade, low and middle income countries, shrimp

## Abstract

Antimicrobial resistance (AMR) is a growing threat to global public health, and the overuse of antibiotics in animals has been identified as a major risk factor. With high levels of international trade and direct connectivity to the aquatic environment, shrimp aquaculture may play a role in global AMR dissemination. The vast majority of shrimp production occurs in low‐ and middle‐income countries, where antibiotic quality and usage is widely unregulated, and where the integration of aquaculture with family livelihoods offers many opportunities for human, animal and environmental bacteria to come into close contact. Furthermore, in shrimp growing areas, untreated waste is often directly eliminated into local water sources. These risks are very different to many other major internationally‐traded aquaculture commodities, such as salmon, which is produced in higher income countries where there are greater levels of regulation and well‐established management practices. Assessing the true scale of the risk of AMR dissemination in the shrimp industry is a considerable challenge, not least because obtaining reliable data on antibiotic usage is very difficult. Combating the risks associated with AMR dissemination is also challenging due to the increasing trend towards intensification and its associated disease burden, and because many farmers currently have no alternatives to antibiotics for preventing crop failure. In this review, we critically assess the potential risks the shrimp industry poses to AMR dissemination. We also discuss some of the possible risk mitigation strategies that could be considered by the shrimp industry as it strives for a more sustainable future in production.

## Introduction

Antimicrobial resistance (AMR) is the term used to describe microbial organisms that can resist the effects of drugs and chemicals designed to kill them. It is now widely regarded one of the greatest risks to human and animal health, and the current rate of spread is such that by 2050 an estimated 10 million people globally will die from resistant infections, with an associated economic cost of £60 trillion (O'Neill 2016). For many years, research into AMR was principally focused on human health, but more recently, much interest has been directed to the agricultural and environmental sectors, due to the intrinsic interlinkages between humans, animals and the environment (the ‘One Health’ notion). Furthermore, almost 70% of total antibiotic use is in livestock and this consumption is projected to rise by 67% by 2030 (Van Boeckel *et al*. 2015). This is a major concern since the overuse of antibiotics has been identified as the single most important factor driving the rise in resistant infections (CDC, 2013). Although antibiotic usage in humans in high‐income countries appears to be gradually reducing (Klein *et al*. 2018), it remains high in some food production sectors, most notably poultry and swine (Van Boeckel *et al*. 2015). In low and middle income countries (LMICs), antibiotic consumption in both humans and animals is increasing (Van Boeckel *et al*. 2015; Klein *et al*. 2018), although precise data is often difficult to obtain since usage is widely unregulated and indiscriminate, with antibiotics freely available ‘over the counter’ and little capacity for enforcing regulations and surveillance. Controlling contagion, through improving sanitation, access to clean water and governance, has also been shown as key to controlling AMR dissemination (Collignon *et al*. 2018), and since these factors are frequently associated with LMICs, this further emphasizes the significant risks of antibiotic usage in animal production in these countries.

Aquaculture (the farming of aquatic organisms) is considered one of the most sustainable sources of animal protein and is the fastest growing food sector, yet in order to meet global demand, production must further expand by 50% by 2050 (FAO, 2014). Currently, aquaculture accounts for around 10% of all animal protein consumed globally, with this figure expected to increase by 50% by 2030 (The World Bank, 2013). In 2016, 93% of aquaculture production took place in LMICs (89% in Asia; FAO, 2016a), and although much of the produce is consumed domestically, there is a huge international export market. As in agriculture, aquaculture is thought to have high levels of antibiotic use, although exact figures for much of the industry are hard to confidently establish due to the lack of surveillance. In terms of risk to AMR dissemination, aquaculture is of particular concern in terms of the propensity for emergence, persistence and transmission within the aqueous environment, which is already known to be an important reservoir of clinically relevant antimicrobial resistance genes (Marti *et al*. 2014). In addition, the relative lack of infrastructure in terms of waste treatment in many LMICs means that any treatments (including antibiotics) and microbial matter (including resistant bacteria) from ponds are often released directly into the aquatic environment. Transmission to people may also be enhanced through direct contact with pond water and livestock; an estimated 18 million people work within the primary aquaculture industry globally (FAO, 2016b). Despite these significant risk factors, aquaculture has thus far received relatively little attention in terms of infectious disease and AMR research.

In this review, we evaluate current understanding of the role of the shrimp aquaculture industry in the emergence and dissemination of AMR. Many other forms of aquaculture, notably finfish, share many of the same risks for AMR (Henriksson *et al*. 2017), but we focus on shrimp aquaculture (used collectively to refer to the farming of shrimp and prawn species) for the following reasons. Firstly, shrimp has a large, growing, global market and is traded extensively around the world. Secondly, the farming of crustaceans brings somewhat different risks and challenges compared to fish species, which in the context of AMR mainly relates to the fact that shrimp do not have an acquired immune system and can therefore be more susceptible to pathogens, often leading to a greater need for antimicrobial treatments (Smith *et al*. 2003; Hauton & Smith 2007). Thirdly, the more primitive immune system in shrimp does not respond to vaccination, which in the fin fish industry has paid great dividends in terms of reducing disease and antimicrobial usage (Sommerset *et al*. 2005; Gudding & Van Muiswinkel 2013). Finally, most shrimp production (87% in 2016) occurs in Asia (FAO, 2016a) and this is an area that has been identified as posing a very significant risk to the global burden of AMR (Chereau *et al*. 2017). In this review, we assess the existing evidence for the emergence and dissemination of AMR in this sector, and review the drivers behind antimicrobial use in shrimp aquaculture worldwide. We also discuss possible risk mitigation strategies, aimed at promoting the prudent use of antibiotics and/or reducing the necessity for them, in order to increase the sustainability of the shrimp industry and minimize its contribution to the growing global AMR problem.

## The global shrimp industry

**Figure 1:**
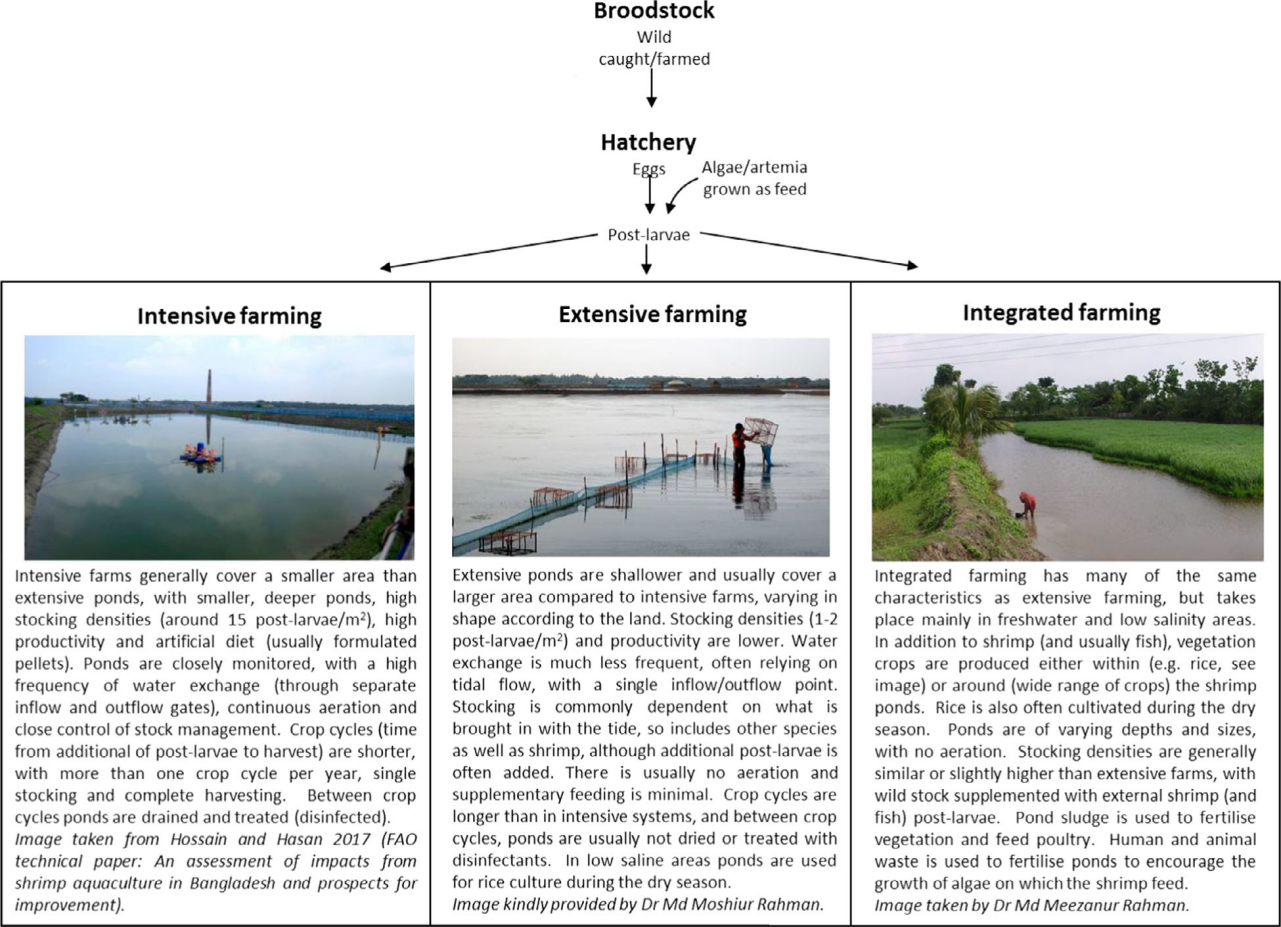
Shrimp farming. Typical shrimp farming process, showing characteristics of the different types of production: intensive, extensive and integrated. Images are of ponds in Bangladesh (credits shown).

In 2016, 67% of global shrimp production was farmed and 33% wild‐captured (FAO, 2016a). Marine shrimp farming makes up the vast majority of production, and mainly involves two penaeid shrimp species, *Penaeus vannamei* (Whiteleg shrimp) and *Penaeus monodon* (Giant tiger prawn). The shrimp industry began to develop on a large scale in coastal areas of South East Asia in the 1970s, and although *Peneaus monodon* was the preferred species for many years, its susceptibility to disease has led to a preference for farming *Litopenaeus vannamei*, which has constituted the largest shrimp industry growth. Freshwater prawn farming has also been gradually increasing, and here the two main species are *Macrobrachium rosenbergii* (Giant river prawn) *Macrobrachium nipponense* (Oriental river prawn; Fig. S1). Shrimp farming has been a traditional way of life for many years, through trapping larvae from rivers or seas and cultivating them. A large proportion of shrimp farmers across the globe remain as small holders, with extensive pond systems (large ponds, with low animal densities), and shrimp farming is often integrated with other types of crops and livestock production, with a view to making the most efficient usage of the land and minimising the economic risks associated with monoculture and disease (Fig. 1). However, in many established shrimp‐producing countries, including Thailand, Vietnam and China, intensive shrimp farming is increasing. In many cases, this includes the use of enclosed recirculating aquaculture systems (RAS) as they require less water and have improved biosecurity in order to reduce disease risk. Intensification of shrimp production has largely been possible due to the adoption of new technologies, such as improved feed formulations and the hatchery production of specific pathogen‐free (SPF) post‐larvae from breeding adults (broodstock; Kumar & Engle 2016). Broodstock are typically obtained from the wild but a reduction in numbers and increased prevalence of disease in wild stock has led to the development of broodstock domestication in some countries, which commonly offer SPF animals (mainly *Litopenaeus vannamei*) that are certified as free from a number of common diseases.

One of the main disadvantages of captive broodstock breeding programmes is that, without very careful management, a reduced genetic diversity can result, increasing the risks associated with disease susceptibility. Disease is a significant constraint on the development and sustainability of shrimp and prawn culture and has had devastating impacts on entire geographical regions of the industry. Shrimp are affected by a range of bacterial, viral, fungal and parasitic pathogens and until recently the highest profile problems have been caused by viral diseases, notably White Spot Syndrome Virus (WSSV); cumulative losses to this pathogen alone were estimated in 2012 to be more than USD 6 billion (Lightner *et al*. 2012). Bacterial diseases are also a major problem. For example, it is now recognized that acute hepatopancreatic necrosis disease (AHPND), is associated with strains of bacterial *Vibrio* species carrying an insect toxin plasmid (Han *et al*. 2015; Restrepo *et al*. 2018). Infections cause up to 100% mortality in post‐larvae shrimp, and losses due to this pathogen were estimated at more than USD 1 billion in Asia at the end of 2013 (FAO, 2013). This increasing disease burden not only threatens the national GDP of these LMICs but also has a huge impact on the livelihoods of those who depend on subsistence farming. To illustrate this, an infection in Vietnam in 2006 resulted in the death of 50% of the 2 billion shrimp post‐larvae cultivated, and this affected an estimated 14,000 households (Anh *et al*. 2010).

**Figure 2:**
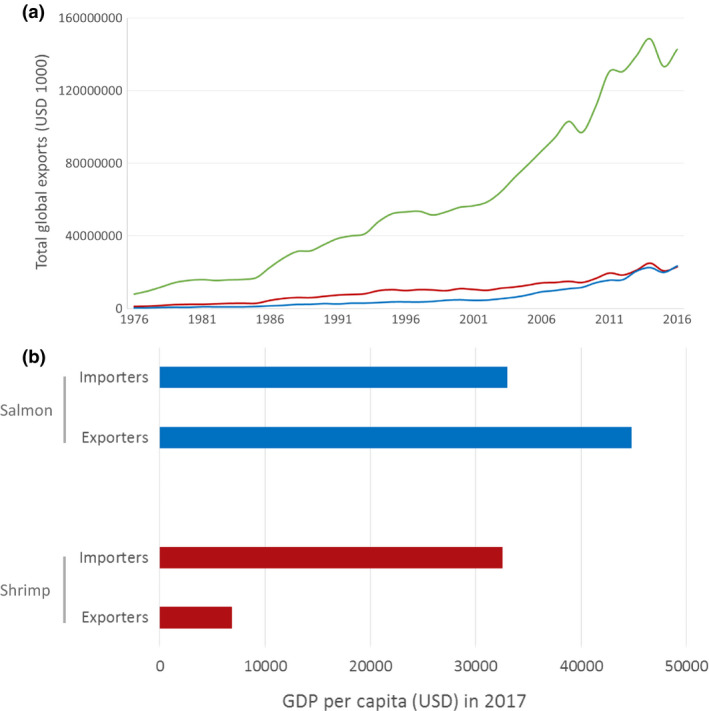
The global shrimp industry. (a) Shrimp and salmon are the highest internationally‐traded seafood commodities by value. In 2016, salmon overtook shrimp as the highest value traded commodity. Data, obtained from the FAO's FishStat J Global Fishery and Aquaculture Commodity Statistics Dataset, shows the total global market value of exports over time. (b) Weighted average GDP per capita of the top 10 shrimp and salmon exporting and importing countries. Weighted averages were calculated by taking the sum of imports/exports (data from the FAO's FishStat J Global Fishery and Aquaculture Commodity Statistics Dataset) for the top 10 countries and calculating each country's fraction of this. This fraction was then multiplied by the GDP per capita for that country (World Bank databank; 2015 data) to obtain the weighted average score. Total aquaculture (green); Shrimp (red); Salmon (blue).

**Figure 3:**
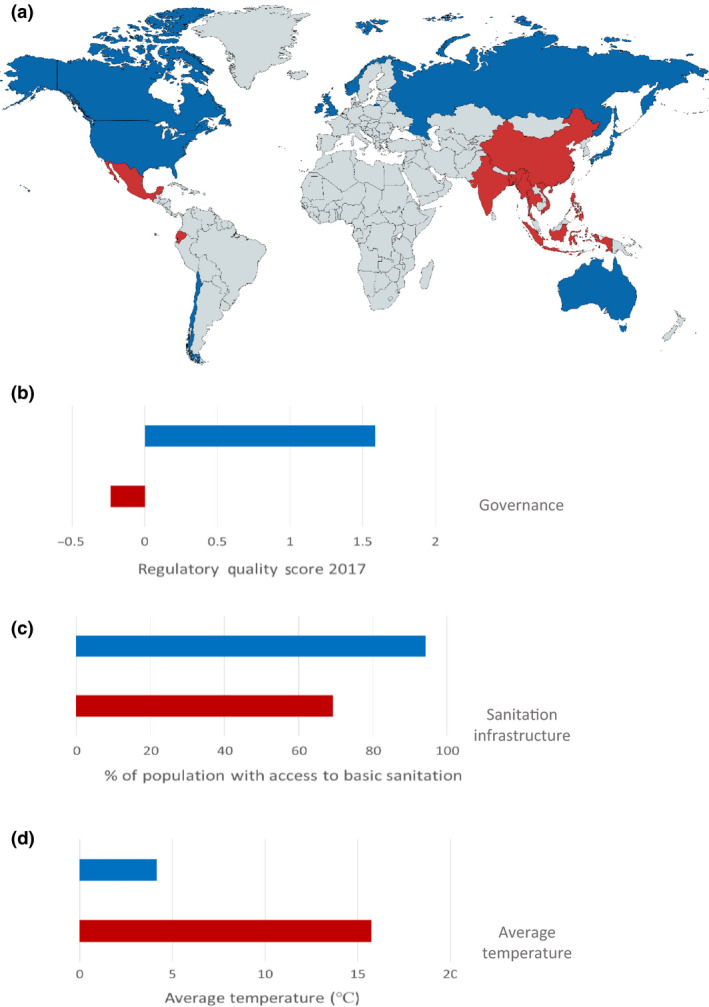
Selected comparisons between the top 10 shrimp and salmon producing countries. (a) Global geographical distributions of the top 10 shrimp (red) and salmon (blue) producing countries. Data obtained from the FAO's FishStat J Global Fishery and Aquaculture Production Statistics Dataset were used to produce the map with www.mapchart.net. (b–d) Differences between shrimp (red) and salmon (blue) producing countries in terms of (b) governance (taken as mean Regulatory Quality Score in 2017; regulatory quality is one of the six dimensions of governance used in the World Governance Indicators Project: http://info.worldbank.org/governance/WGI/#home), (c) sanitation infrastructure (World Bank Databank 2015 data: People using at least basic sanitation services (% of population)) and (d) climate: average monthly temperature between 1991–2015 (World Bank Climate Change Knowledge Portal).

Despite the high disease burden, shrimp was the most highly traded food commodity by value for many years, until it was overtaken in 2016 by salmon (Fig. 2a). In this year, these two products alone represented 32.5% of the aquaculture export industry (USD 23.5 billion exports for salmon and USD 22.9 billion for shrimp; FAO, 2016a). Although the international market value of these commodities is very similar, the nature of these industries differs greatly. Salmon are exported and traded between high‐income countries (HICs), whereas the majority of shrimp production takes place in LMICs and is exported to HICs (Figs 2b and S2). The growth of both the shrimp and salmon industries has been largely supported by the intensification of production, however, the increased levels of disease associated with this intensification has placed an ever‐growing importance on the containment of both known and emerging pathogens. Between 1987 and 2013, the Norwegian aquaculture industry (which is predominantly salmon production) increased from 300,000 tonnes to 1.2 million tonnes; the use of vaccination against major pathogens and the availability of water of high quality is considered to have supported the containment of the disease through this intensification, since antibiotic use was reduced by 99% during this same period (Norwegian Ministry of Health and Care Services, 2015). In contrast, the reduction in the rate of growth of the shrimp industry is largely due to problems with disease, and antimicrobial agents (e.g. disinfectants, fungicides) have become an integral part of many shrimp farming‐operating procedures. Improved water quality, better hygiene and stricter farm biosecurity measures would undoubtedly go a long way to addressing the problems with disease in shrimp production, and therefore reduce the need for antibiotic usage. Levels of sanitation infrastructure and governance in the main shrimp‐producing countries are also very low (Figs 3a–c and S3), and, without the option of vaccination, there is limited scope for prophylaxis. Due to the nature of shrimp farming, most shrimp‐producing countries also have a warmer climate that lends itself to greater pathogenic burden and an increased number of vectors such as insects and migratory birds, which have been shown to play an important role in both disease and AMR dissemination (Fig. 3d; Ahlstrom *et al*. 2018; Onwugamba *et al*. 2018).

### Antibiotic usage in shrimp aquaculture

A wide range of antibiotics and other antimicrobials, including heavy metals, fungicides and antiparasitics, are used in shrimp aquaculture, with many reports describing the use of clinically relevant antibiotics, particularly in hatcheries (Holmstrom *et al*. 2003; Uddin & Kader 2006; Thuy *et al*. 2011; Mostafa Shamsuzzaman & Kumar Biswas 2012; Ali *et al*. 2016; Chi *et al*. 2017; Hinchliffe *et al*. 2018). However, as is the case in most LMICs, accurate antimicrobial sales and usage data are difficult to obtain since antibiotics are freely available over the counter without a veterinary prescription, and can be of varying quality (Tran *et al*. 2018). Many shrimp farmers do not have easy access to professionals or facilities for accurate disease diagnosis, and obtain advice on treatments from farm supply shops, neighbouring farmers, government representatives, non‐governmental organizations such as Worldfish (https://www.worldfishcenter.org/) or drug manufacturers/vendors, who are also known to provide financial incentives (Pham *et al*. 2015). Collating information on antibiotic sales and usage is also difficult, due to considerable differences between farm types, sizes, species, climate, geography, disease risk, water supply, etc., even within one country. Further, antibiotic usage may fluctuate greatly from year to year, even in the same regions, due to climate and disease outbreaks. This high level of variation in antibiotic usage is reflected in inconsistent reports from farmer surveys (Lyle‐Fritch *et al*. 2006; Thuy *et al*. 2011; Rico *et al*. 2013; Tuševljak *et al*. 2013; Ali *et al*. 2016; Liu *et al*. 2017; Thi Kim Chi *et al*. 2017), and this situation is familiar across the aquaculture world, with reviews on antibiotic usage in general aquaculture demonstrating the scale and complexity of this issue (Vignesh *et al*. 2011; Done *et al*. 2016; Muziasari *et al*. 2016; Mo *et al*. 2017; Lozano *et al*. 2018).

Antibiotics are typically applied by mixing with feed or, less commonly, directly to the water. The direct application of antibiotics only to the individual animals requiring treatment (therapy) is not practical, so treatments are typically considered to be metaphylactic (where the whole population of animals, including both infected and uninfected animals are treated). True prophylactic use in aquaculture, where the animals are treated in the absence of disease, is considered rare in farms but more common in hatcheries (Smith 2008, 2012; Zhang *et al*. 2011). A recent study on Bangladesh shrimp hatcheries reported the use of up to 80 kg antibiotics per production cycle in a single hatchery (Hinchliffe *et al*. 2018).

The limited scope for immunoprophylaxis results in antibiotics currently being the most common method for preventing and treating disease in shrimp aquaculture, despite many of the most devastating diseases in recent years having been viral. Thus, although we cannot estimate the potential losses saved by the use of antimicrobials, antibiotic usage in shrimp aquaculture is likely to be inappropriate and inefficient, due to limited diagnostic capacity, poor regulation of quality and usage and poor application (in terms of antibiotic selection, doses, methods, timing, etc.). Generally, antibiotic usage (and antimicrobial use more widely) is thought to be higher in intensive rearing systems where there is a greater need for chemical agents to clean and maintain shrimp pond hygiene, as well as to prevent or treat disease.

## Antimicrobial resistance in the shrimp industry

The ability of AMR genes to pass between bacteria, a process known as horizontal gene transfer, or HGT, is thought to underlie the rapid rise in resistant pathogens seen across the globe (Martinez *et al*. 2015). This process can occur between unrelated bacterial species (Li *et al*. 2018), meaning that resistance genes (which confer antibiotic resistance to the bacteria carrying them) present in non‐pathogenic, environmental bacteria (many species of which are unidentified) can be transferred to animal or human pathogenic bacteria and pose a threat to animal and human health. This risk is most likely to occur in environments where non‐pathogenic and pathogenic bacteria are brought into close contact, such as animal/human guts and environments where animal/human waste is present, such as waste water treatment plants, agricultural soils and in surface water run‐off from land applied with organic fertilization. Resistance gene dissemination is further exacerbated by the presence of antimicrobial compounds, which select for the expression and transfer of resistance genes, even at low concentrations (Wistrand‐Yuen *et al*. 2018). There is evidence to support the transfer of AMR genes from clinically relevant pathogens to aquatic organisms, with studies demonstrating that fish and shellfish pathogens have acquired resistance genes within mobile genetic elements (MGEs; segments of DNA that are easily transferred from one bacterium to another, e.g. transposons and plasmids) with very high similarity to those previously recovered from clinical bacterial isolates, demonstrating likely shared recent origins (McIntosh *et al*. 2008). An increasing number of publications have described the genetic basis of AMR in aquaculture environments, and a comprehensive review on this subject has been recently published (see Miller & Harbottle, 2018). Thus, the presence of resistance genes in MGEs in fish pathogens (including zoonotic pathogens) demonstrates the potential for AMR dissemination to animal/human/environmental bacteria through HGT.

**Figure 4:**
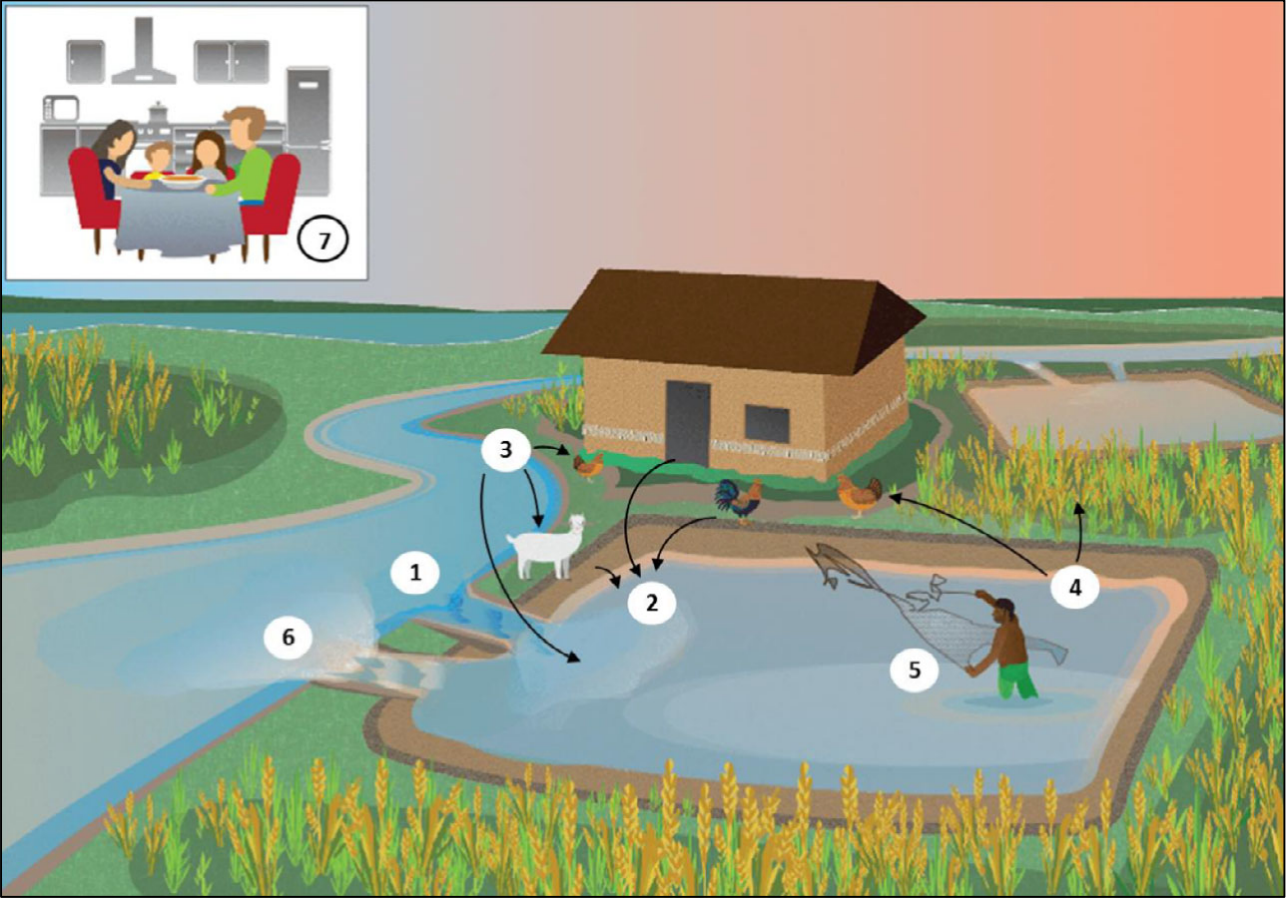
Ways in which shrimp farming poses a potential risk to AMR dissemination for a typical integrated small‐scale shrimp farm. 1. Fresh water is sourced from local water sources and mixed with saline water, in order to reach the desired salinity. These water supplies may be polluted with antibiotics, resistant bacteria and human/animal pathogens. 2. Human and animal waste, containing human/animal pathogens, is often used to fertilize the pond to encourage the growth of algae on which the shrimp feed. 3. Unregulated use of antibiotics, which can be added directly to the pond water and/or to the animals/humans living there. 4. Pond sediment/sludge containing antibiotics and resistant bacteria/pathogens is used as fertilizer for chicken feed and crops, both of which are eaten by the humans. 5. Humans have direct contact with pond water, sediment/sludge and shrimp/other animals. 6. Waste (water and sediment) is released directly back into local water sources, often untreated. 7. Shrimp (and associated bacteria/antibiotic residues) are transported and consumed internationally.

Considering the size and international nature of the shrimp industry, there is very little information on AMR in shrimp farming environments. This is surprising as these farms are potential AMR dissemination hotspots for many reasons: stocking densities are often very high, which brings with it associated high bacterial densities and raised stress levels for shrimp, which in turn makes the stock more susceptible to infection; high levels of disease incidence; the widespread usage of chemicals; the release of untreated waste into the local environment; and high levels of unprotected human contact with water/animals. The large quantities of shrimp that are then transported and consumed both domestically and internationally make it likely that this industry poses a significant risk to global AMR dissemination (Fig. 4). Although many of these risk factors can be applied to a large proportion of the aquaculture industry in general, one of the AMR risks that is particularly pertinent to shrimp farming is the high level of traditional integrated farming practices, where human and livestock/poultry waste is used to fertilize ponds in order to promote algal growth within the pond which the shrimp then feed on. This is not only common but also promoted by many governments as being more environmentally and economically sustainable, however it increases the prevalence of human and zoonotic pathogens in the shrimp pond. Furthermore, the heavy use of antibiotics in livestock and poultry increases the levels and variety of antibiotic residues present in the pond, increasing the selection pressure. In these farming systems, sludge (sediment) from ponds is often used to feed the animals or fertilize vegetable or rice fields, which are subsequently also used for human and animal consumption. Thus, an environment is created which favours the emergence and spread of AMR genes, and this is supported by reports identifying high levels of resistance in these systems (Suzuki & Hoa 2012). Indeed, in one study in an integrated fish farming, it was shown that there was a significant increase in resistance across a crop cycle, from levels of 1–5% of bacteria to 80–100% in 2 months, and particularly high levels of resistance in human‐associated *Enterococcus* bacteria (Petersen *et al*. 2002).

The impact of resistant infections on shrimp health is largely unknown, since few farmers have access to diagnostic services to identify the pathogen causing the disease, let alone the resistance levels. In 1994, mass mortality of shrimp larvae due to antimicrobial resistance was reported in Indian hatcheries, where 70–90% of larvae died from infection with strains of *Vibrio harveyi* that were highly resistant to a number of drugs, two of which (cotrimoxazole and chloramphenicol) had been used prophylactically. The lack of resistant *Vibrio* in the input sea water and shrimp eggs led the authors to conclude that the long‐term use of these drugs had led to a build‐up of resistant bacteria in the larval rearing tanks (Karunasagar *et al*. 1994). This was also supported by a separate study by the same researchers, who tested water samples from six hatcheries in India and found that larval tanks were predominantly populated by *Vibrio* species, with very high levels of resistance to many different antibiotics (Otta *et al*. 2001).

**Figure 5:**
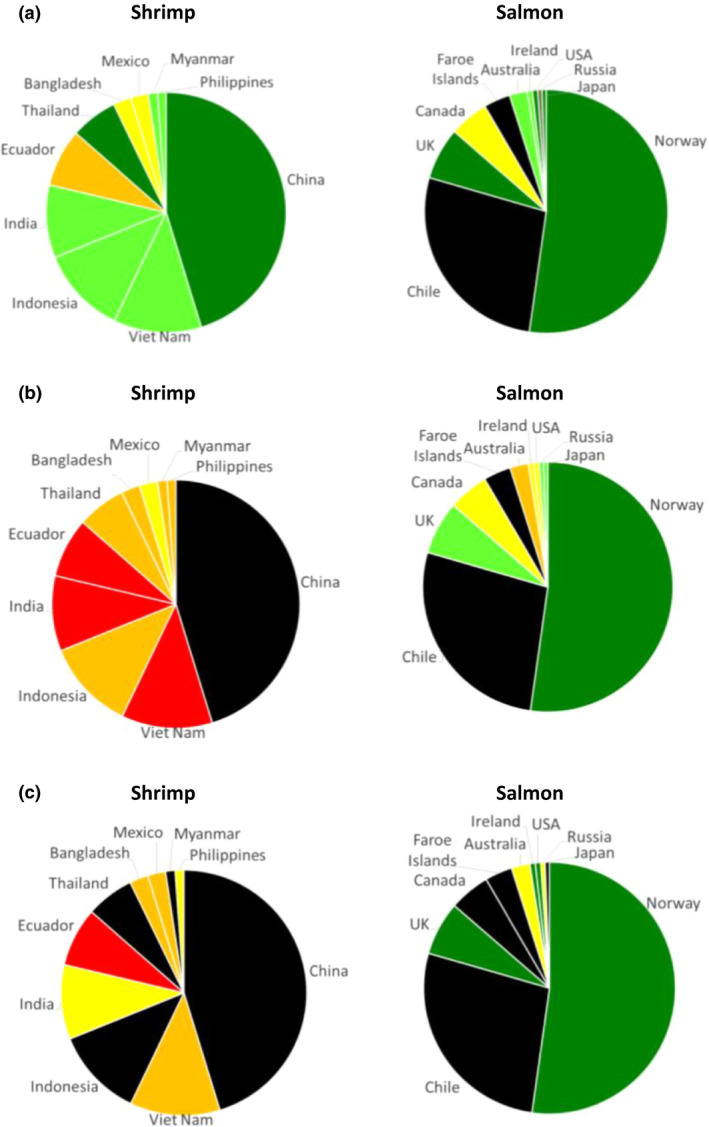
Progress on AMR governance. Data from the WHO AMR self‐assessment survey, in which a multi‐sectoral group from each country is asked to submit annual responses to a questionnaire on AMR and antimicrobial use, in order to monitor progress and identify areas for action. Data shows country responses for the top 10 shrimp‐ and salmon‐producing countries (year two data, 2017–2018, published on 18th July 2018) for the topics below. Countries rate themselves from A (no action being taken; red) to E (best practice; dark green). Responses for each category are shown, taken directly from the self‐assessment survey. Black indicates no response given. Pie chart proportions indicate relative levels of production (in metric tonnes) for each country. OIE: World Organisation for Animal Health. (a) No response (black); No national AMR action plan (red); National AMR action plan under development (orange); National AMR action plan developed (yellow); National AMR action plan approved by government that reflects Global Action Plan objectives, with an operational plan and monitoring arrangements (light green); National AMR action plan has funding sources identified, is being implemented and has relevant sectors involved with a defined monitoring and evaluation process in place) (dark green). (b) No response (black); No national plan or system for monitoring sales/use of antimicrobials in animals (red); Plan agreed for monitoring quantities of antimicrobials sold for/used in animals, based on OIE standards (orange); Data collected and reported on total quantity of antimicrobials sold for/used in animals and their intended type of use (therapeutic or growth promotion) (yellow); On a regular basis, data is collected and reported to the OIE on the total quantity of antimicrobials sold for/used in animals nationally, by antimicrobial (light green); class, by species (aquatic or terrestrial), method of administration, and by type of use (therapeutic or growth promotion) (dark green) Data on antimicrobials used under veterinary supervision in animals are available at farm level, for individual animal species). (c) No response (black); No national plan for a system of monitoring of AMR is available (red); National plan for monitoring AMR but capacity (including laboratory) for surveillance and reporting data on AMR is lacking (orange); Some AMR data is collected locally but may not use a standardised approach and lacks national coordination and/or quality management (yellow); Priority pathogenic/ commensal bacterial species have been identified for surveillance (light green). Data systematically collected and reported on levels of resistance in at least 2 of those bacterial species, involving a laboratory that follows quality management processes, e.g. proficiency testing; National system of surveillance of AMR established for priority pathogens and for relevant commensal bacteria which follows quality assurance processes in line with intergovernmental standards (dark green). Laboratories that report for AMR surveillance follow quality assurance processes).

With the tripartite agreement from the WHO, FAO and OIE focused on addressing the problem of AMR on a global scale, there is an increasing drive towards better regulation of antimicrobial usage. In line with this, all of the top 10 shrimp‐producing countries had AMR action plans under development as of July 2018, according to the WHO AMR self‐assessment survey, which has set out to collect data from each country annually in order to monitor and evaluate the global AMR action plan (Fig. 5). Implementation of these action plans is, however, more of a challenge, and progress towards this is far less advanced (Table S1). When compared to the salmon industry, shrimp‐producing countries show much less progress in developing national monitoring systems for both antimicrobial use and AMR in animals and the environment (Fig. 5 and Table S1). Over a decade ago, the FAO's consultation on ‘Antimicrobial Use in Aquaculture and Antimicrobial Resistance’ identified that there was a need for more national and regional data on AMR, antimicrobial residues and antimicrobial usage, as well as for more knowledge on the spread of AMR genes from aquatic and fish bacteria to human pathogens (WHO, FAO and OIE, 2006). This is still the case today, with only a few studies having looked at AMR in the shrimp farming industry. A summary of the published studies describing evidence for AMR in bacteria associated with farmed shrimp species is provided in Table S2. It is very difficult to compare the results of the different studies due to considerable variation in the type of sample analysed (e.g. farmed/retail shrimp, pond water, sediment samples), the shrimp species involved, bacterial species characterized, antibiotics assessed and the methods used to characterize AMR. These variations can sometimes be explained by examining the purpose of the study: those that investigated AMR in human pathogens associated with shrimp (commonly *Vibrio* and *Aeromonas* species) often tested the susceptibility to antibiotics that are used to treat human infections, whereas those that aimed to assess the effect of AMR on the efficacy of the main antibiotics used in aquaculture usually tested the susceptibility to those antibiotics. Thus, the direct comparison between studies is not possible due to the differences in methods and interpretative criteria deployed. Another important issue is that 10 of the 37 studies highlighted in Table S2 that tested for phenotypic resistance did not state whether they used standardized methods and interpretative criteria for susceptibility testing (CLSI or EUCAST, see section below on *Standardization of surveillance of antimicrobial usage and AMR in shrimp aquaculture*). Further, of those that did, many cited the standard but did not provide evidence that they had followed the well‐defined interpretative criteria. The use of standardized methods is essential to ensure results between different studies are properly comparable (Schwarz *et al*. 2010). It is also important to avoid conflation acquired with intrinsic resistance, since many organisms have natural low susceptibility to certain classes of antibiotics. For instance, many *Vibrio* are intrinsically resistant to first generation penicillins (Chiou *et al*. 2015). A number of human pathogens are also intrinsically resistant to colistin, and, like many of the polymyxins, colistin binds to the surface of plastics and does not diffuse well through agar, meaning that results from many of the typical disc diffusion methods are meaningless (World Health Organization, 2018).

**Figure 6:**
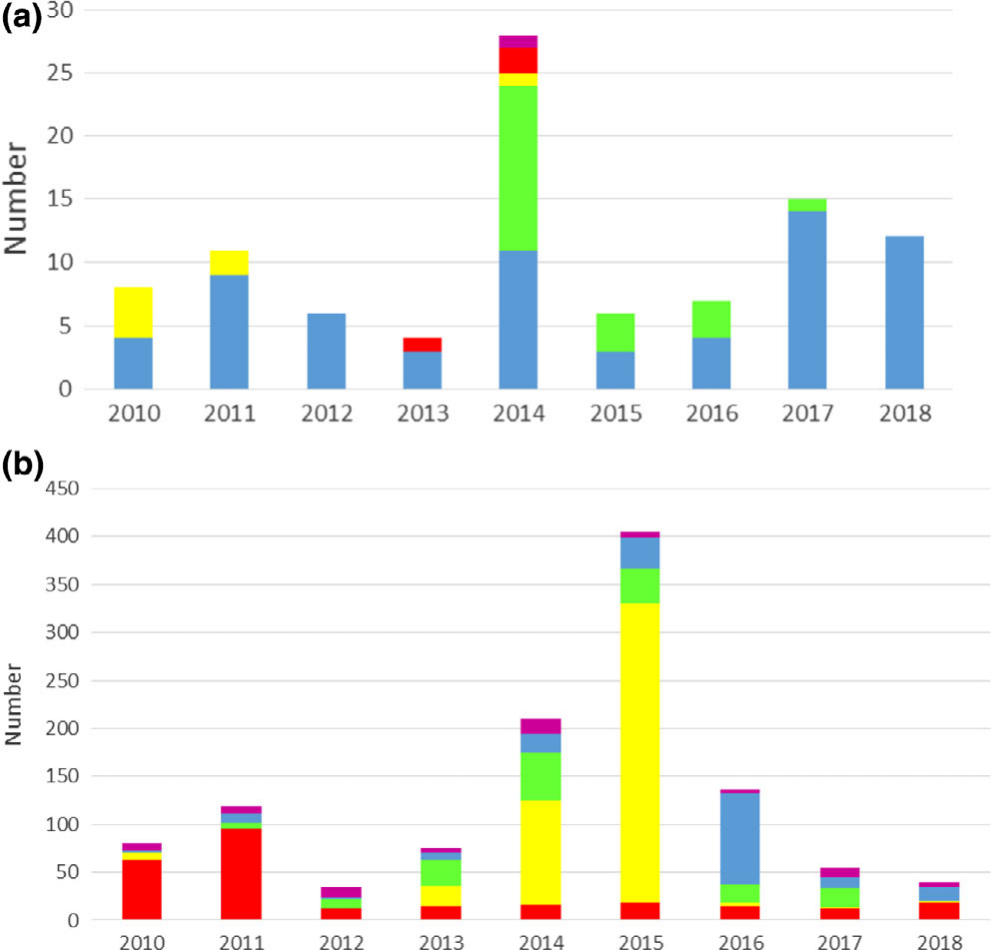
Shrimp export rejections due to the presence of residues of prohibited antibiotics or those exceeding the maximum residual levels permitted. Number of border rejections due to antibiotic residue hazards in (a) EU (data taken from EU's Rapid Alert System for Food and Feed portal on 03/12/2018, analysing search results from 01/01/2010 to 31/10/2018, filtering only shrimp border rejections due to antibiotic residues). India (blue); Vietnam (green); Bangladesh (yellow); China (red); Myanmar (purple). (b) USA (data taken from FDA's Refusal Entry Database, from 01/01/2010 – 31/10/2018, filtering only shrimp border rejections due to antibiotic residues. Data on total number of samples tested each year were not readily available from either database. China (red); Malaysia (yellow); Vietnam (green); India (blue); Others (purple)

### Dissemination of resistance through shrimp farms and hatcheries

High levels of antimicrobial resistant bacteria have been reported in the environment local to shrimp farms (Sundaramanickam *et al*. 2015; Rocha *et al*. 2016), and AMR genes have been shown to persist in the environment close to aquaculture sites even after antibiotic usage has ceased (Tamminen *et al*. 2011). This is particularly concerning since many of the pathogens present in shrimp ponds and hatcheries (e.g. *Vibrio* and *Aeromonas* species) are endemic to the surrounding marine and estuarine environments, meaning that resistant bacteria released into these environments have the potential to become the predominant strains. This risk is compounded by the high levels of antibiotic pollution in the environment surrounding shrimp aquaculture facilities, with growing evidence of the role of farms themselves in this pollution, through the release of water and pond sediments (Akinbowale *et al*. 2006; Hoa *et al*. 2011; Hossain *et al*. 2017, 2018; Lai *et al*. 2018). Inflowing local water sources can also be heavily polluted with antimicrobial compounds and resistant bacteria. For example, in China it was reported that the AMR genes present in shrimp farm input water correlated with that of the pond water, both in their abundance and temporal patterns (Su *et al*. 2017). Resistant bacteria can also enter shrimp farms through products added to the ponds, such as shrimp feed and probiotics. In intensive and semi‐intensive shrimp farming, pellet feeds containing animal‐ and plant‐based products are common, however their composition varies and antibiotic compounds are often added in order to preserve the feed in hot climates. To our knowledge, no studies have investigated the carriage of AMR genes in shrimp feed, although multidrug‐resistant pathogens have been detected in fish feed, with one study reporting 132 unique AMR genes and four mobile genetic elements in five fishmeal products (Han *et al*. 2017; Antunes *et al*. 2018; Novais *et al*. 2018). Extensive shrimp farming is more commonly associated with home‐made feeds mixed with antibiotics, however one survey of Vietnamese farmers found that many did not know the appropriate method of use, or dose, of the antibiotics incorporated, and sometimes even did not know the name of the antibiotic being used (Le & Munekage 2004). Concerns have also been raised over the accuracy and quality of the probiotics sold to shrimp (and other aquaculture) farmers, with studies reporting bacterial content different from those listed on the label, and carrying resistance to multiple antimicrobials (Noor Uddin *et al*. 2015; Uma & Rebecca 2018). Unconsumed feed and probiotics also contribute to antibiotic‐and AMR‐rich waste feed and faeces released into the local environment, where they may be taken up by wild organisms (Thuy *et al*. 2011). For example, it is thought that 95% of consumed oxytetracycline is released into the surrounding environment through faeces (Serrano 2005). Due to the high levels of untreated waste containing antimicrobial residues and resistant bacteria discharged by shrimp farming into the local environment, it is also possible that human populations living downstream of these facilities may be directly exposed. A recent study by Shen *et al*. (2018) looked at levels of *Escherichia coli* carrying the *mcr‐1* gene conferring resistance to colistin in stool samples from more than 5000 individuals across China, and found that higher levels of *mcr‐1* correlated with living in aquaculture‐intense areas. The use of colistin in animals is now banned in China, but used to be orally administered to pigs and poultry and is poorly absorbed, thus the authors suggested that these levels could be due to faecal contamination of the aquatic environment, where colistin is highly stable.

Given the nature of the shrimp farming environment, many shrimp farms are at, or close to, sea level, and flooding poses a huge threat to farmers’ livelihoods. It also provides a means by which resistant bacteria and antibiotic residues present in the ponds and aquatic environment can be disseminated over an enormous area very quickly. Increasing global temperatures have also been suggested to play a role in increasing AMR (MacFadden *et al*. 2018), presumably due to increased temperatures favouring bacterial growth. Indeed, a higher prevalence of *V. parahaemolyticus* was detected in shrimp sold at Chinese domestic markets during the summer months (Yang *et al*. 2017).

Another important factor contributing towards AMR emergence and dissemination in the shrimp farming environment is co‐selection with other antimicrobials, such as anti‐fungicides, anti‐parasitics and heavy metals (Seiler & Berendonk 2012). If these are present, selection for resistance genes can take place at lower antibiotic concentrations (Murray *et al*. 2018). High levels of heavy metals, commonly lead, cadmium, chromium, copper and zinc, have been detected in shrimp and pond water (Seiler & Berendonk 2012; He *et al*. 2016; Sarkar *et al*. 2016). Many heavy metals adsorb to particulate matter and are concentrated in the pond bed sediments where the shrimp reside and feed. Antibiotics are also known to persist in pond sediments as when adsorbed to them they degrade more slowly (Thuy *et al*. 2011). Thus, shrimp pond sediments may be a hub for AMR emergence and dissemination, and indeed these have been shown to contain higher levels of antibiotics and antibiotic‐resistant bacteria than pond water (Le & Munekage 2004; Le *et al*. 2005). Other sources of aquaculture pollution also promote the growth of micro‐organisms, and therefore increase the risk of AMR dissemination, in the local environment, such as organic enrichment with nitrates or phosphates (Anh *et al*. 2010).

One of the main direct routes for AMR dissemination to humans in the shrimp industry is likely to be through human contact with resistant bacteria present in the farm environment. Compared to salmon farming, shrimp farming occurs in countries where aquaculture is labour‐intensive; ten times more people are employed per tonne of production in the main shrimp‐producing country (China; 78 people per 1000 tonnes) compared to the main salmon‐producing country (Norway; 6 people per 1000 tonnes; FAO, 2016b). Thus, human exposure levels are considered to be very high in shrimp farming, largely due to a greater prevalence of traditional farming methods. For example, small‐scale farm workers frequently wade through ponds to feed, assess and harvest animals, and most live in close proximity to ponds and other animals on the farm who are exposed to the same pathogens and AMR genes. Many more people are also exposed through shrimp processing and onward sale. In agricultural settings, a number of studies have now shown that humans in direct contact with animals (e.g. farm workers) have increased levels of AMR genes and resistant bacteria associated with the farm animals and the antibiotics they were being exposed to (Marshall & Levy 2011). Reports of this in aquaculture are very limited, with one study showing that people working within Korean aquaculture had a higher faecal carriage of resistant bacteria than control restaurant employees (Shin & Cho 2013), and a second identifying quinolone resistance genes in marine bacteria in a Chilean salmon aquaculture area that were identical to those in urinary tract *E. coli* pathogens from humans living close by, suggestive of HGT (Tomova *et al*. 2015). There are also bacterial infections associated with shrimp handling, for example, fish‐handler's disease, commonly caused by infection of cuts in the skin with *Mycobacterium* species, which are rarely serious but can develop into deeper tissue infection or sepsis. Although there are no reports of resistance being a problem in these infections, they provide a route by which resistance genes can potentially enter the human body.

Human consumption of shrimp is another route by which AMR genes from shrimp can be transferred into human pathogens, since any resistant bacteria associated with shrimp are brought into close contact with human gut bacteria (Hoelzer *et al*. 2017). A number of human pathogens are associated with shrimp, such as *Vibrio parahaemolyicus*, one of the most important causes of food‐borne bacterial infectious diarrhoea in South East Asia (Nair *et al*. 2007; Li *et al*. 2014), and *Vibrio vulnificus,* a serious human pathogen carrying a very high case fatality rate (Jones & Oliver 2009). Resistance genes have been detected in shrimp products destined for human consumption (Table S2), with one study associating high levels of the colistin resistance *mcr‐1* gene with consumption of meat, pork, mutton and aquaculture products (Shen *et al*. 2018). Consumption of resistant strains of these bacteria not only poses a threat to humans through their inability to be treated, but also raises the levels of resistance genes in the gut, increasing the likelihood of dissemination.

## Risk mitigation strategies

In order to be effective, measures put in place aimed at limiting the risks of AMR dissemination must be appropriate for each geographical, social, political and cultural context (World Health Organization, 2015; Kakkar *et al*. 2018). There are a number of key areas that the industry as a whole, or at least individual countries or areas within them, could consider. As with all AMR action plans, the principal means by which the risk of AMR dissemination in the shrimp industry might be mitigated is through reducing contagion and promoting the prudent use of antimicrobials. Indeed, controlling contagion is essential for the shrimp industry irrespective of the risks of AMR, due to the ever‐increasing burden of disease that threatens the future of the industry and the livelihoods of those within it.

### Regulate antibiotic sales/usage

For effective prevention and better control over the use of antimicrobial agents in aquaculture, national and international level regulatory frameworks on the sales, use and quality of antimicrobial agents are required. All of the top 10 shrimp‐producing countries do have regulations in place for the use of antibiotics in aquaculture, but there is a huge disconnect between regulation and enforcement. In an ideal world, veterinarians, or other licensed fish health professionals, would prescribe antimicrobials, and licensing would be enforced and guided by qualified fisheries and animal health professionals. This is not the case in most shrimp‐producing countries, and imports to the EU and US are regularly rejected due to testing positive for prohibited antibiotic residues or residues above the maximum residue levels (MRLs; Fig. 5). For example, between 1st October 2014 and 30th September 2015, 32% of sampled shrimp imports from peninsular Malaysia to the US tested positive for chloramphenicol and/or nitrofuran residues, antimicrobial compounds that are banned for use in food production in Malaysia (Fig. 6). This led to the FDA imposing an Import Alert on shrimp products from Malaysia in April 2016 (FDA Import Alert #16‐136). Similarly, there has recently been a growing concern within the EU over the level of antibiotic use in the Indian shrimp farming, and, accordingly, the rate of testing has been increased from 10 to 50 per cent (Behera 2018). However, although export restrictions undoubtedly help to reduce antibiotic usage, they only detect antibiotic usage immediately prior to harvesting, and do not apply pressure to hatcheries and shrimp destined for domestic markets. It is also worth noting that the balance of trade is shifting from a bipolar Global South to North, to South‐South trading, upsetting the current trade‐related and buyer‐led forms of regulation (Belton *et al*. 2018). Despite significant progress in the development of AMR action plans, a significant barrier to reducing antibiotic usage in the major shrimp‐producing countries is the lack of resources to implement the regulations that are already in place.

### Alternatives to antimicrobials

There is currently limited scope for prophylaxis through vaccination in shrimp, due to their inability to raise an adaptive immune response, although the increasing evidence for innate immune memory may offer future possibilities (Milutinović & Kurtz 2016). Alternative options have focussed on stimulating shrimp immunity and maintaining a healthy shrimp and pond microbiome (a term given to describe the suite of microorganisms in a particular environment), with pond water bacterioplankton composition having been suggested as a potential indicator of shrimp health status (Zhang *et al*. 2014). A number of immune stimulants are now available for use in shrimp (Barman & Nen 2015), although there is limited data on their effectiveness. Probiotics are widely used, and prebiotics have also shown some efficacy in shrimp. As an example, *Litopenaeus vannamei* fed on a diet with supplementary poly‐b‐hydroxybutyrate (PHB) had increased growth and feed conversion rates, and showed improved immunity (Duan *et al*. 2017). Similar positive results were seen with PHB in *Macrobrachium rosenbergii* larvae (Nhan *et al*. 2010). Many farmers also reportedly use herbal medicines (Chi *et al*. 2017), and these have been shown to exhibit antimicrobial effects. Examples here include two essential oils from the Madagascan plant, *Cinnamosma fragrans*, which had effects similar to erythromycin in lowering bacterial count and increasing survival rates of *Penaeus monodon* larvae in hatcheries (Randrianarivelo *et al*. 2010). The identity of the active ingredients of many herbal remedies, however, are unknown and some may be toxic to humans or the environment. In addition, it is highly likely that resistance will ultimately develop to any antimicrobial compound present.

One of the most promising alternative treatment options to replace antibiotics is phage therapy. Bacteriophages (referred to as ‘phages’) are viruses that infect and kill bacteria, so theoretically they could be used to treat infections and reduce levels of resistant bacteria. However, in order for this to be effective, the infecting/resistant pathogen must be identified, and this diagnostic capacity is not widely available in the shrimp industry. One option that has been suggested for shrimp aquaculture is to target bacteria which are known to colonize shrimp ponds and cause infections in both shrimp and humans, for example, phages against *Vibrio harveyi* have been isolated from shrimp hatcheries and shown to increase survival of larvae. In one study, phage therapy over a period of 17 days increased the survival of *Penaeus mondon* larvae from 17 to 86%, whereas antibiotic treatment survival rates were 40% (Vinod *et al*. 2006). However, it must be noted that phages have also been shown to be vectors for the dissemination of AMR genes, since they can transfer pieces of DNA from one bacterial host to another (Subirats *et al*. 2016). Other compounds specifically targeting *Vibrio* bacteria are also available and could be potential treatments for shrimp *Vibrio* infections, as well as more general methods such as quorum‐sensing inhibitors that interfere with bacterial cell–cell communication (Defoirdt *et al*. 2011).

### Improved disease diagnosis

Accurate diagnosis of disease allows for the selection of appropriate antimicrobials, and avoidance of antibiotics critical for use in human health. This would prevent the use of antibiotics for viral infections, but the administration of medicines in these circumstances may in fact be reasonable given the suite of possible pathogen interactions at any one time. For example, WSSV infection has been shown to increase susceptibility to other bacterial pathogens (Phuoc *et al*. 2008). Yet, many shrimp‐producing countries do not have the resources to provide farmers with easy and affordable access to veterinary officers or diagnostic facilities, and so farmers have no choice but to self‐diagnose and self‐treat. Better diagnostic support and access would allow farmers to receive advice not only on the best treatment, but also on the best application method, dose and length of treatment, all of which would help to reduce unnecessary antimicrobial usage. In line with this, our own work with WorldFish in Bangladesh is supporting the development of in‐country skills sets for accurate pathogen and disease diagnosis. We are doing this through facilitating the use of a combination of molecular (largely PCR‐based methods, but also more comprehensive sequencing approaches, e.g. MinION sequencing) and histopathology techniques. The associated research in this programme of work is also seeking to better understand the assemblages of microorganisms associated with shrimp infections and disease states – the so‐called pathobiome, as many diseases in shrimp (and finfish) aquaculture appear to be polysyndromic in nature and are not caused by a single pathogen (Stentiford *et al*. 2017 ).

Advances in next generation‐sequencing technologies have transformed human health infectious disease diagnosis, and offer much potential for transfer to the aquaculture sector. Whole genome‐sequencing (WGS) of bacterial isolates not only facilitates accurate diagnosis, but also allows for improved monitoring of disease epidemiology, as well as the detection and surveillance of AMR genes and associated mobile genetic elements (Bayliss *et al*. 2017). In addition, research into shrimp transcriptomics shows potential for applications in health monitoring and the development of immune stimulants or treatments to common infections (Rodriguez‐Anaya *et al*. 2018). As more of the shrimp industry moves towards selective breeding of broodstock, these new technologies also offer much scope for genomic‐assisted selection, for example, through identification of genes encoding greater tolerance to specific pathogens or stress (Guppy *et al*. 2018). With such potential impact, it is no surprise that much of the current scientific research into shrimp aquaculture is focusing on the application of these technologies, although the capacity to implement any outcomes from this research remains limited in most shrimp‐producing countries, as most laboratories do not have the expertise, staffing or equipment to conduct such studies. Laboratory capacity and expertise is improving, however, through initiatives such as the UK government‐funded Fleming Fund (https://www.flemingfund.org/), and technological advances such as the development of the portable MinION sequencer (https://nanoporetech.com/products/minion) are increasing the accessibility of these applications to the shrimp industry. Further, pond‐side Genedrive platform PCR‐based diagnostics, that are straightforward enough for farmers to use, are also becoming a reality, and these have recently been deployed in commercial farm settings across regions in Thailand (Minardi *et al*. 2019).

### Improved hygiene and farm management

Better management practices (BMPs) for shrimp farming are often based upon the FAO's Code of Conduct for Responsible Fisheries (FAO, 1995), with the aim of standardising management practices. Usually, BMPs are adapted for the various types of shrimp production (e.g. intensive/extensive), and thereby aim to reduce the risk of disease and environmental degradation specific to these production systems. For example, in terms of antimicrobial usage, large vertically integrated companies may be able to put in place advanced biosecurity systems to limit the spread of disease both within and between their ponds, but this relative control may be offset by higher stocking densities and greater perceived risks relative to loss values. Both may act as drivers for higher disease risk and greater usage of antimicrobials. In contrast, subsistence farmers may find control much harder to achieve. In these cases, farmers may also be less risk averse, especially if practising polyculture where losses to shrimp crop may be offset by other products (e.g. rice, fish; Hinchliffe *et al*. 2018; Little *et al*. 2018). In all production types, proper management of the aquaculture environment in the terms of parameters such as feed rates, dissolved oxygen, stocking densities, movement restrictions, water treatment and biological control could go a long way in avoiding excessive use of antimicrobial agents. Thus, one of the routes to reducing unnecessary antimicrobial usage is to encourage farmers to adopt BMPs. A recent report by Suzuki & Hoang Nam 2018 reported that in Vietnamese shrimp farming, which consists mainly of small‐scale farms, receiving training and appropriate information increased BMP adoption, and that BMP adoption reduced disease outbreaks.

### Increased species diversity

A general shift in shrimp farming from *Penaeus mondon* to *Litopenaeus vannamei* in the last decade is considered to have helped the expansion of the industry. This is because *Litopenaeus vannamei* has a higher feed conversion rate and can tolerate a wider range of salinities, allowing inland farming where there is lower salinity. Importantly for antibiotic usage, it also has a higher tolerance to disease, with higher hatchery survival rates (Kumar & Engle 2016). Since pathogens often affect one species more than another (e.g. *Penaeus monodon* is thought to be more susceptible to WSSV than *Litopenaeus vannamei*; Tuyen *et al*. 2014), it could be argued that the industry would benefit from a wider variety of species, and a lower disease burden would reduce the need for antimicrobials. Interestingly, genetically improved species have been successfully introduced to tilapia farming (Kumar & Engle 2016), so there could be scope for selected breeding of new shrimp species to reduce the disease burden in shrimp.

### Better regulation of feed and probiotic manufacture and sales

The use of medicated feed is widespread in aquaculture. Avoiding overuse of feed is thus a relatively straightforward way to reduce environmental contamination with antimicrobial residues and resistant bacteria, since up to 30% of feed is unconsumed (Cabello *et al*. 2013). Inappetence is a typical symptom of infections in aquatic animals, so providing medicated feed can be of questionable benefit unless very carefully managed (Ranjan *et al*. 2017). Regulations on feed used in aquaculture are in place in some countries, for example the Vietnamese National Fisheries Quality Assurance and Veterinary Directorate (NAVIQAVED) attempts to test and control feed quality across the industry. However, the large number of feeds available on the market makes this very difficult, with more than 100 types of feed produced domestically and many more imported from across the world (Anh *et al*. 2010).

Probiotics are now widely used in shrimp aquaculture to stimulate immunity and improve feed digestion and water quality, despite scepticism from the scientific community over their efficacy. A few studies have suggested that administration of probiotics to *Litopenaeus vannamei* post‐larvae can improve growth and feed efficiency, increase expression of immune related genes and even protect against infection by WSSV (Chai *et al*. 2016; Miandare *et al*. 2016). However, reports identifying discrepancies in probiotic contents and labelling, and the presence of resistant bacteria, show the need for better regulation of their contents and improvements in the information provided for the farmer on their specific purpose, dosage and correct application measures (Noor Uddin *et al*. 2015). Thus, as for medicated feed, avoiding excess use of probiotics is necessary to reduce any risk of contamination with unknown and potentially resistant bacteria.

### Farmer education

Many of the major shrimp‐producing countries offer a range of courses on aquaculture management for farmers to attend, ranging from government/non‐governmental organisation workshops to university degrees. However, surveys conducted on farmers’ knowledge of antibiotic use have suggested that they have a limited understanding about the purpose of antibiotics, their appropriate use, dose and method of application (Holmstrom *et al*. 2003; Mostafa Shamsuzzaman & Kumar Biswas 2012; Pham *et al*. 2015). For example, one survey of Vietnamese farmers found that, despite 45% of respondents believing that antibiotics had no effect, 86% still used them, and 12 of the 23 antibiotics being used were listed as critically important by the WHO (Pham *et al*. 2015). In a separate study, 27% of Thai shrimp farmers surveyed were using antibiotics to treat viral diseases such as white spot syndrome (Holmstrom *et al*. 2003). In line with these reports, the WHO AMR self‐assessment survey found that training and professional education on AMR in the farming sector was very poor in the top 10 shrimp farming countries, although the high‐income salmon farming countries had much to improve on too (Table S1).

### Certification

Aquaculture certification and labelling programmes have become the primary tool to address sustainability issues in farmed seafood, which includes promoting prudent antimicrobial usage. Such schemes are already in place, with the majority of all certified aquaculture production covered through the Aquaculture Stewardship Council (ASC), Global Aquaculture Alliance Best Aquaculture Practice (GAABAP), Global G.A.P. and Friend of the Sea. To increase audit efficiency and reduce farmer administrative workload, the three leading aquaculture certification programmes, ASC, GAABAP and Global G.A.P. have now drafted and agreed combined checklists for farms that seek more than one certificate, however the growth in the number of certification schemes has increased confusion. As a result, the Global Sustainable Seafood Initiative (GSSI) has been created, tasked with developing a global benchmarking tool to measure and compare certification schemes and standards performance. Despite this progress on certification, one of its main disadvantages is that it is largely focused on the export market, and has limited influence on the production of shrimp destined for the domestic market and hatcheries, where certification is less valued. Further, the costs associated with becoming certified, and the requirement for book keeping and auditing, are more accessible to larger farms, whereas the majority of shrimp farmers, in many countries, are family‐run smallholders.

### Standardization of surveillance of antimicrobial usage and AMR in shrimp aquaculture

Over a decade ago, the WHO/FAO/OIE's consultation on ‘Antimicrobial Use in Aquaculture and Antimicrobial Resistance’ identified that there was a need for both more national and regional data on AMR, antimicrobial residues and antimicrobial usage, as well as for more knowledge on the spread of AMR genes from aquatic and fish bacteria to human pathogens (World Health Organization, Food and Agriculture Organization of the United Nations and World Organization for Animal Health, 2006). Improved surveillance has been identified as key to reducing antimicrobial usage across all human and animal health sectors, and is an integral part of national AMR action plans. Surveillance of antimicrobial usage and resistance would cover the entire shrimp industry and would not be restricted to products destined for export. However, like most LMICs, the main shrimp‐producing countries are still at a very early stage in the development of any type of AMR/AMU surveillance system (Fig. 5), and effective surveillance will be very difficult to achieve without first gaining tighter control of antibiotic sales.

Standardization of the methods used to both measure sensitivity to antibiotics and then interpret the resultant antimicrobial sensitivity testing (AST) data (Alderman & Smith 2001; World Organization for Animal Health, 2018) is also crucial. It is strongly recommended that Clinical and Laboratory Standards Institutes (CLSI; or other equivalent international standards, e.g. EUCAST) are used for this type of testing. It is also very important that appropriate quality assurance processes are in place to ensure the integrity of AST data. For bacteria isolated from aquatic animals, the creation of CLSI standards for antimicrobial disk diffusion testing and determination of minimal inhibitory concentrations by broth micro‐ and microdilution are important steps in this process (CLSI, 2006, 2014a,2014b). It is recognized that further work is required here to develop these provisional standards, particularly with the types of pathogens that affect tropical species such as shrimp.

There are major efforts underway (e.g. via the UK Government‐funded Fleming Fund) to support LMICs to improve their laboratory capacity for the surveillance of AMR and antibiotic usage. Although most of this will understandably be used to improve surveillance in clinical settings, it is also recognized that this should encompass animal (including aquaculture) production in these countries. As an example, the Fleming Fund and UK Government have recently provided funding to support the development of a new Food and Agriculture Organisation of the United Nations (FAO) designated International Reference Centre for AMR, run through the UK agencies of the Department for Environment, Food and Rural Affairs ‐ Veterinary Medicines Directorate (VMD), Centre for Environment Fisheries and Aquaculture Science (CEFAS) and the Animal and Plant Health Agency (APHA). The centre will seek to improve the balance of the one‐health approach and take the steps needed to improve AMR surveillance in the animal and environmental sectors. Specifically, the centre will provide technical assistance, training and quality assurance to support an increase in AMR surveillance in low‐ and middle‐income countries across Asia and Africa.

## Conclusions

There is a growing movement to reduce the use of antimicrobials in food animals in general, but many of the practical solutions put forward, such as increasing the price of antibiotics and capping the amount used per animal, rely on governments having the means to implement and enforce regulations and assess their efficacy, which is not the case in most shrimp‐producing countries (Van Boeckel *et al*. 2017). One of the main barriers to better regulation is that the Competent Authorities responsible for their implementation in many shrimp‐producing countries are very understaffed and under resourced. As a result, there is often a paucity of officials compared to the number of farms, and a lack of the knowledge required for accurate diagnosis of the disease conditions. This makes long term programmes (e.g. surveillance) difficult to implement and become effective. To this end, a phased approach to creating the surveillance networks required for LMIC governments to effectively achieve reductions in inappropriate antimicrobial usage has recently been suggested (Schar *et al*. 2018). Another barrier to effective antibiotic sales and usage regulation and surveillance in many shrimp‐producing countries is the informal medicines supply network, in which the sale of substandard and counterfeit antibiotics is a problem (Zellweger *et al*. 2017). It is unrealistic, and unfair, to expect farmers in LMICs to reduce antibiotic usage without major investments in farm hygiene, biosecurity and associated diagnostic/veterinary services (UN Interagency Coordination Group on Antimicrobial Resistance, 2019). In many shrimp‐producing countries, the majority of diagnostic and prescriptive services are provided by a largely untrained and unregulated paraveterinary sector, since there are simply not enough trained veterinary officers (Hinchliffe 2019).

Thus, the infrastructure for the central control of the regulatory, diagnostic and surveillance systems does not exist or is insufficient in many shrimp‐producing countries. Adopting as models the systems that work in high income countries may not be practical in the timescales that are required to reduce the global burden of AMR (Cox *et al*. 2017). Solutions could therefore look towards more self‐regulatory processes. This could include a greater involvement of extension officers, such as those employed by WorldFish and other international non‐governmental organisations, for delivering training and educational programmes (https://www.worldfishcenter.org/). Raising farmer awareness of better management practices should also improve farm hygiene and biosecurity, thereby reducing contagion and AMR dissemination. More generally, increased community awareness of AMR and the risks of antibiotic misuse could promote a degree of self‐policing, of both farmers and the local antibiotic suppliers, which in shrimp farming communities are often untrained local farm shop owners (Hinchliffe 2019). Aquaculture could usefully learn from the human health sector, which is facing the same issues in LMICs in terms of lack of resources for regulation and surveillance of antibiotic sales and usage. In some LMICs, antibiotic stewardship programmes in the human drug sector have been focusing on the drug suppliers, for example the accredited drug dispensing outlet (ADDO) programme, which began in Tanzania and has now been extended to other African countries and Bangladesh (Rutta *et al*. 2015). This programme of accreditation for local drug shops, which includes training and signing up to codes of conduct, has seen significant improvements in dispensing practices, although it has been noted that future efforts should also include public engagement, in order to reduce public demand for these drugs (Embrey *et al*. 2016).

Since AMR and inappropriate antibiotic use are true ‘One Health’ issues, that do not respect differences in human/animal sector boundaries, it seems sensible that these sectors both learn from and work with each other, in order to effect maximal understanding and impact. In the context of AMR, local actions have international consequences, thus national governments, international agencies/organisations and the private sector need to co‐ordinate their efforts, knowledge and resources if we are to reduce the risk that the shrimp industry poses to global AMR dissemination. This review has highlighted some of the socioeconomic, political and cultural aspects of this risk, and has outlined some of the major challenges that must be overcome in order to mitigate it. In doing so, we have demonstrated the paucity of information available on the use of antimicrobials, the prevalence of AMR in shrimp and human pathogens, as well as on the types of resistance mechanisms carried by these species in shrimp aquaculture. As a result, it is currently very difficult to accurately assess the risks posed by the use of antimicrobials in this rapidly growing sector, which includes a wide range of production systems at the interface between the aquatic and terrestrial environments. Given the complexity of the shrimp aquaculture environment in terms of potential sources of AMR genes and antibiotic residues, and the means by which these are disseminated, it is therefore vital to gather more available data on AMR and antibiotic sales/usage in shrimp aquaculture in order to establish where future efforts aiming to reduce this are best placed. Equally important, however, is the need for wider public engagement and a better understanding of the social, economic and political limitations to achieving these goals in each country. A co‐ordinated approach, between experts in the aquaculture, clinical, environmental and social disciplines, from both the private and public sectors, will be essential.

## Data availability statement

The research data supporting this publication are provided within this paper.

## Supporting information


**Figure S1.** Shrimp production.
**Figure S2.** Top 10 shrimp and salmon importing and exporting countries in 2016.
**Figure S3.** Top 10 shrimp and salmon producing countries in 2016.
**Table S1.** Selected data from the WHO AMR self‐assessment survey (a subset of which is shown in Fig. 5).

**Table S2.** Evidence for antimicrobial resistance in bacteria associated with farmed shrimp.
