# Supplementary material for: Evaluating antimicrobial resistance in the global shrimp industry
Source: Rev Aquac. 2019 Jul 8;12(2):966–86. doi: 10.1111/raq.12367 (PMC7319481; doi:10.1111/raq.12367)

**Supplementary Figure 1: Shrimp production.** Production (by quantity, in metric tonnes) of the top five farmed shrimp species since the industry began in the 1970s. Data obtained from the FAO's FishStat J Global Fishery and Aquaculture Production Statistics Dataset.

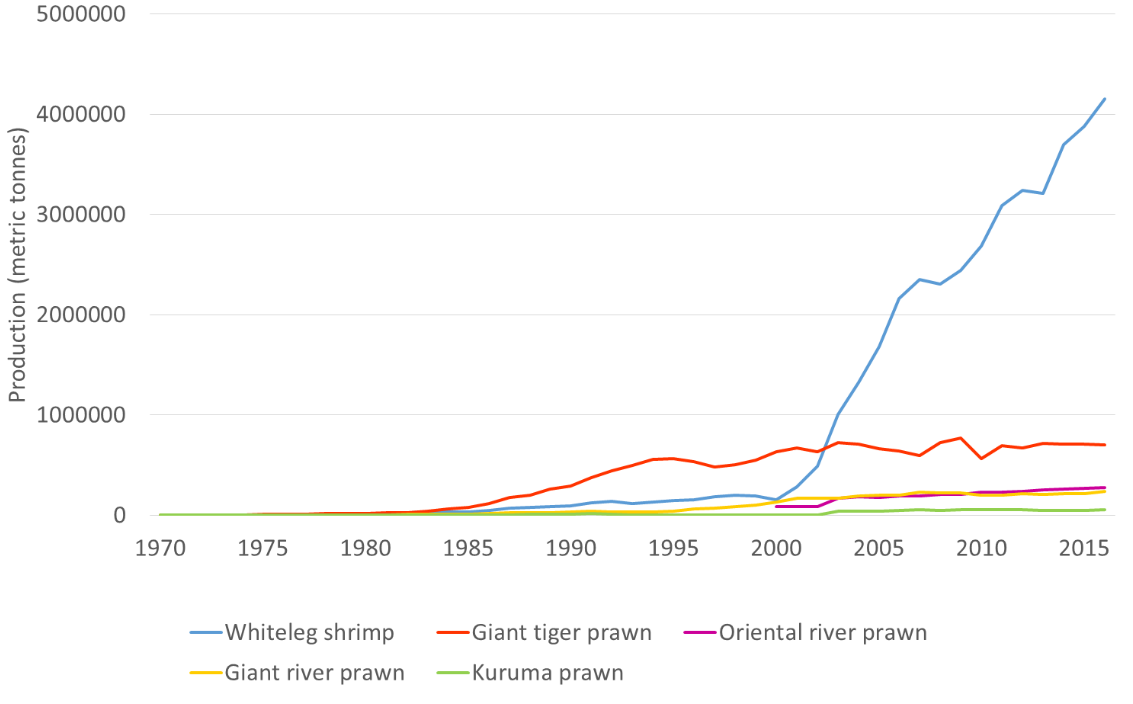

**Supplementary Figure 2: Top 10 shrimp and salmon importing and exporting countries in 2016.** Data obtained from the FAO's FishStat J Global Fishery and Aquaculture Commodity Statistics Dataset. Numbers above bars indicate percentage of total exports/imports.

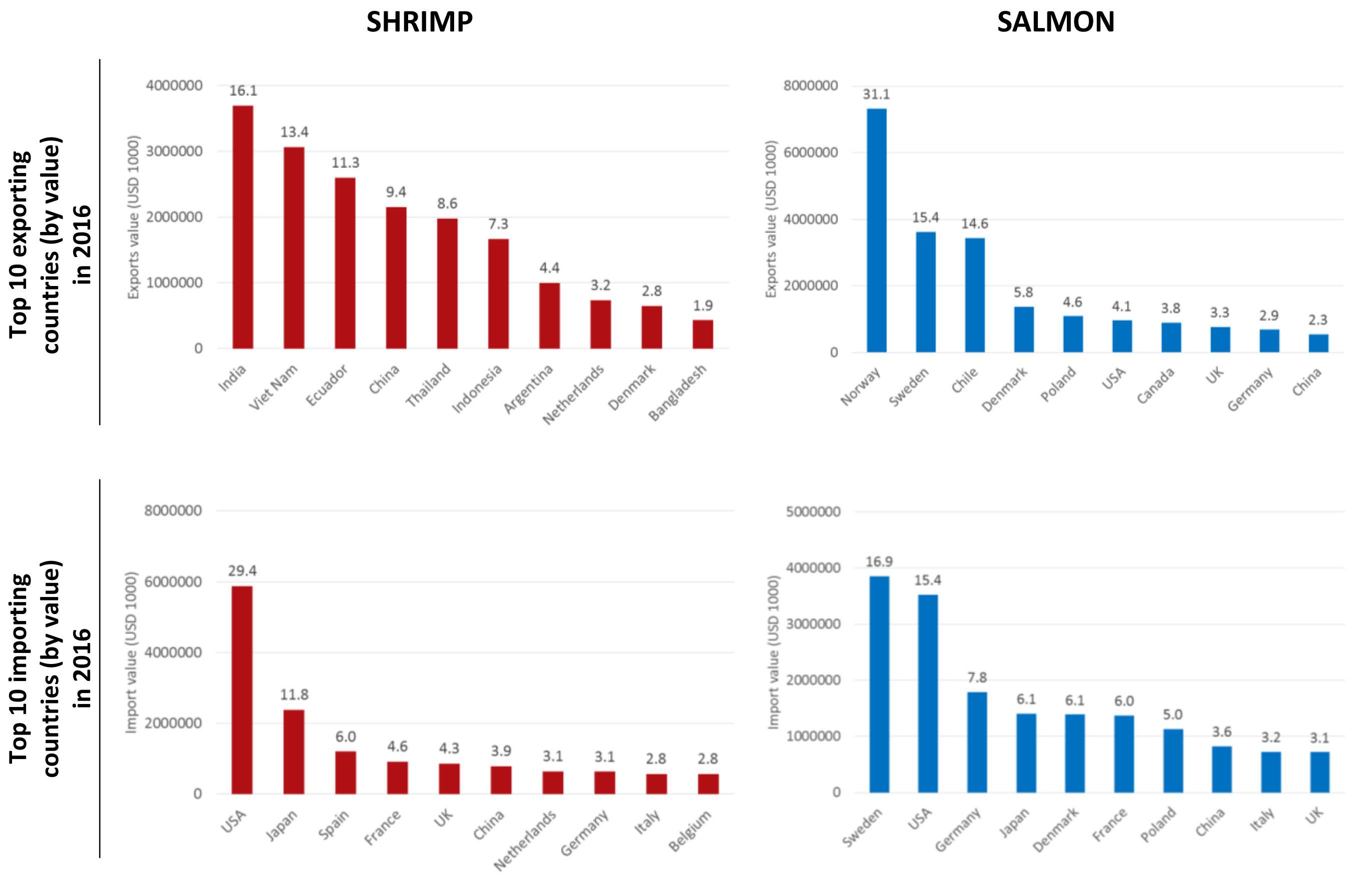

**Supplementary Figure 3: Top 10 shrimp and salmon producing countries in 2016.** Data was obtained from the FAO's FishStat J Global Fishery and Aquaculture Production Statistics Dataset. Numbers above bars indicate percentage of total production.

**SHRIMP**

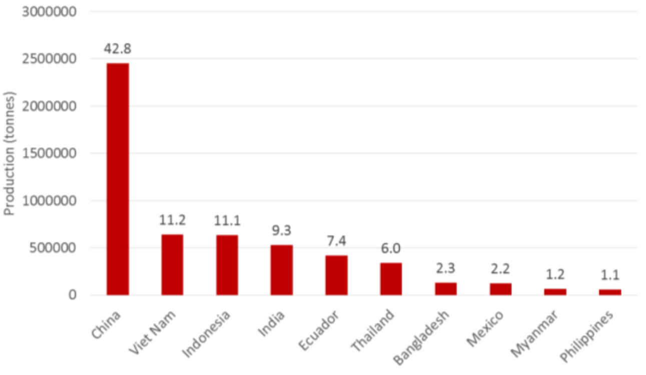


**SALMON**

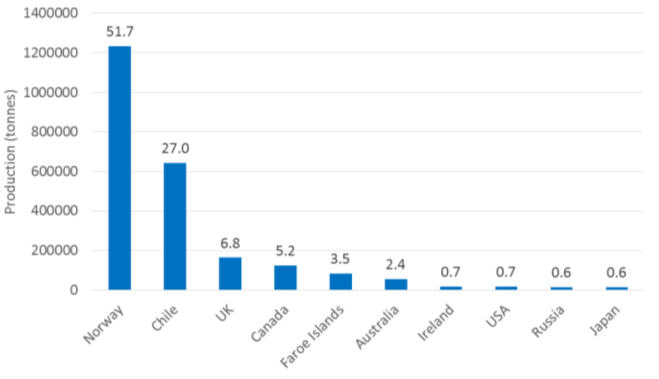

**Supplementary Table 1:** Selected data from the WHO AMR self-assessment survey (a subset of which is shown in Figure 5). Data shows country responses for the top 10 shrimp- and salmon-producing countries (year two data, 2017-18, published on 18th July 2018) for the selected topics below. Countries rate themselves from A (best practice; dark green) to E (no action being taken; red). Black indicates no response given.

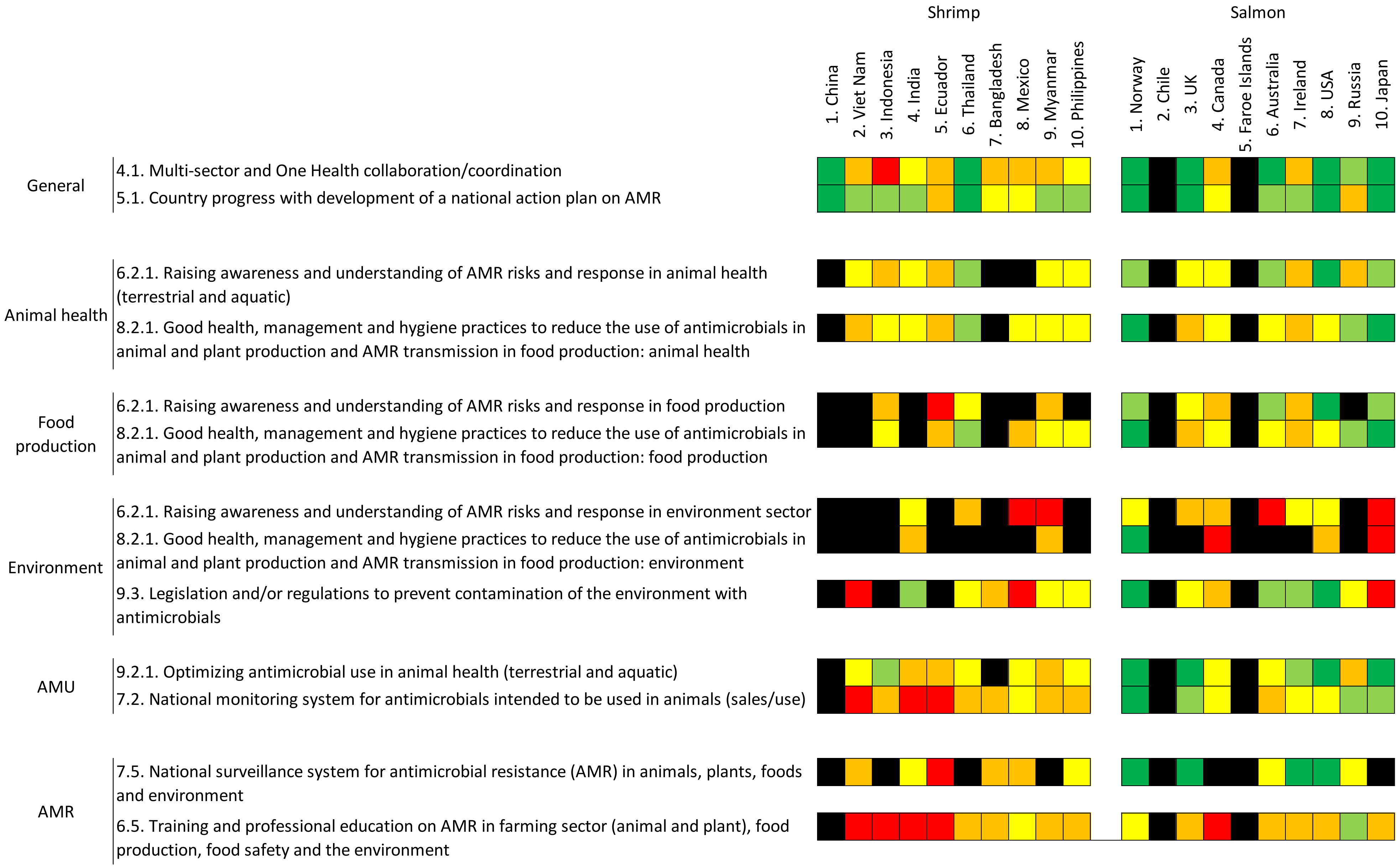

Supplement: Supplementary file 1 — Figure S1. Shrimp production. Figure S2. Top 10 shrimp and salmon importing and exporting countries in 2016. Figure S3. Top 10 shrimp and salmon producing countries in 2016. Table S1. Selected data from the WHO AMR self‐assessment survey (a subset of which is shown in Fig. 5). [file RAQ-12-966-s001.pdf]
