# Supplementary material for: Evaluating antimicrobial resistance in the global shrimp industry
Source: Rev Aquac. 2019 Jul 8;12(2):966–86. doi: 10.1111/raq.12367 (PMC7319481; doi:10.1111/raq.12367)
Supplement: Supplementary file 2 — Table S2. Evidence for antimicrobial resistance in bacteria associated with farmed shrimp. [file RAQ-12-966-s002.pdf]

Supplementary Table 2: Evidence for antimicrobial resistance in bacteria associated with farmed shrimp. List of all known publications that have studied antimicrobial resistance in farmed shrimp: the shrimp species sampled, type of sample (farmed/retail shrimp, pond water/sediment, etc), country of origin, analysis method (†indicates that the publication refers to the use of CLSI standards), bacterial species tested (if looking at isolated strains), resistance detected (in terms of antibiotic class) and molecular mechanism of resistance identified (if applicable). Publications that have studied resistance in more than one species and do not define resistance specifically in shrimp samples have been omitted. Acronyms for antibiotic classes (listed according to WHO classification):

- **Critically important for human health (‡denotes highest priority): CEPH-3/4/5‡** = 3rd/4th/5th generation cephalosporins; **GLY‡** = glycopeptides; **MAC‡** = macrolides; **QUIN‡** = quinolones; **POLY‡** = polymxyins; **AGLY** = aminoglycosides; **ANS** = ansamycins; **CARB** = carbapenems; **MONO** = monobactams; **PEN** = penicillins (natural, aminopenicillins and anti-pseudomonal).
- **Highly important for human health**: **CEPH-1/2** = 1st/2nd generation cephalosporins; **AMPH** = amphenicols; **LIN** = lincosamides; **SUL** = sulphonamides; **TET** = tetracyclines; **APEN** = penicillins (anti-staphylococcal).
- **Important for human health**: **ACYC** = aminocyclitols; **CYPOL** = cyclic polypeptides; **NIT** = nitrofurantoins.

**Reference**	**Species sampled**	**Sample type**	**Country of origin**	**Bacterial species tested (no. isolates)**	**Analysis method**	**Resistance detected (antibiotic class)**	**Molecular mechanism (if determined)**
(Albuquerque Costa *et al.*, 2015)	*L. vannamei*	Farmed shrimp (haemolymph)	Brazil	*Vibrio spp.* (100)	Disc diffusion†	**CEPH-3/4/5‡, MONO, PEN, CEPH-1/2, TET**	
(Arfatahery, Davoodabadi and Abedimohtasab, 2016)	Does not state species	150 wild marine shrimp, 150 farmed shrimp.	Iran	*Staphylococcus aureus* (206)	Disc diffusion†	**AGLY, PEN, TET, APEN**	
(Banerjee *et al.*, 2012)	*L. vannamei*	Farmed shrimp (clinically healthy, muscle & intestine plus pond water)	Malaysia	*Vibrio spp.* (42); *Salmonella spp.* (5)	Disc diffusion†	**MAC‡, PEN, TET, SUL**	
(de Almeida *et al.*, 2017)	*L. vannamei*	Retail shrimp	Brazil	*Enterobacteria* (5)	Disc diffusion†	**CEPH-3/4/5‡, PEN, CEPH-1/2**	
(de Macedo *et al.*, 2016)	*L. vannamei*	Fresh and frozen shrimp	Brazil	*Staphylococci* (17)	Disc diffusion†	**QUIN‡, PEN, TET, APEN**	

(de Melo *et al.*, 2011)	*L. vannamei*	Retail shrimp	Brazil	*V. parahaemolyticus* (10)	Disc diffusion	**AGLY, PEN**	
(De Silva *et al.*, 2018)	*L. vannamei*	Frozen retail shrimp	Korea	*Aeromonas spp.* (44)	Disc diffusion†	**QUIN‡, AGLY, CARB, PEN, CEPH-1/2, TET SUL, APEN**	PCR assays showed presence of *qnrB*, *qnrS*, *tetA*, *tetE*, *aac(6¢)-Ib*, *phAI-IAB*, and *intI1* gene, with 80% integron 1-positive isolates harboring *qacE2*, *dfrA1*, *orfC*, *orfD*, *aadB*, *catB3*, *oxa-10*, and *aadA1* genes.
(Dib *et al.*, 2018)	Red shrimp	Retail shrimp	Algeria	*E. coli* (4)	Disc diffusion†	**CEPH-3/4/5‡, AGLY PEN, CEPH-1/2, AMPH, TET, SUL**	Two strains contained plasmids. *Bla(CTM-X)* gene detected in one by PCR.
(Han *et al.*, 2015)	Not defined	Shrimp	Vietnam, Mexico, India, USA, Philippines, Ecuador and Peru	*V. parahaemolyticus* (78)	Disc diffusion†	**PEN, TET**	Plasmid-mediated *tetB* gene
(He *et al.*, 2015)	*M. ensis*, *M. rosenbergii* and *P. monodon*	Retail shrimp and aquaculture water (does not specify source)	China	*V. cholerae* (42)	Disc diffusion†	**AGLY, ANS, PEN, TET**	
(He *et al.*, 2016)	*L. vannamei*, *M. rosenbergii*, *P. monodon*, and *E. carinicauda*	Retail shrimp	China	*V. parahaemolyticus* (400)	Disc diffusion†	**AGLY, ANS, PEN,** ACYC	
(Hua and Apun, 2013)	*P monodon*	Shrimp, pond water and sediment	Malaysia	*V. parahemolyticus* (140)	Disc diffusion	**QUIN‡, PEN, TET**	
(Jana *et al.*, 2014)	*P. monodon*	Water and sediment samples from ponds before during and after harvest	India	Marine heterotrophs (does not state number of isolates)	Bacterial growth on antibiotic-supplemented media	**PEN, TET, AMPH**	

(Karunasagar *et al.*, 1994)	*P. monodon*	Larvae, eggs, nauplii, sea water, larval tank, algae and artemia	India	Luminous bacteria (does not state number of isolates)	Disc diffusion	**MAC‡, AGLY, AMPH, TET**, NIT	
(Le, Munekage and Kato, 2005)	*P. monodon*	Water and sediment samples from ponds	Vietnam	Heterotrophs (does not state number of isolates)	Bacterial growth on antibiotic-supplemented media	**QUIN‡**, SUL	
(Letchumanan *et al.*, 2015)	*P. indicus* and *S. subnuda*	Retail shrimp	Malaysia	*V. parahaemolyticus* (185)	Disc diffusion†	**CEPH-3/4/5‡, QUIN‡, AGLY, CARB, PEN, AMPH, TET, SUL**	PCR detection of B-lactam (*blaTEM*, *blaSHV*, *blaOXA*), tetracycline (*tetA*, *tetB*, *tetC*, *tetG*), chloramphenicol (*catA1*, *catA2*, *catA3*, *catB3*) and kanamycin (*aphA-3*) resistance genes.
(Molina-Aja *et al.*, 2002)	Peneaid shrimp	Diseased shrimp	Mexico	*Vibrio spp*. (30)	Disc diffusion	**QUIN‡, AGLY, PEN, CEPH-1/2, TET, AMPH**, NIT	
(Nawaz *et al.*, 2012)	*P. monodon*	Imported shrimp (into US)	Thailand	*Klebsiella spp* (67)	Disc diffusion†	**QUIN‡, AGLY, ANS, PEN, TET, SUL, AMPH,** CYPOL	
(Nawaz *et al.*, 2015)	*P. monodon*	Imported shrimp (into US)	Does not state	*E. coli* (105)	Disc diffusion†	**QUIN‡, AGLY, ANS, PEN, TET, AMPH**	PCR detection of mutated *gyrA*, *parA*, *parC*. *dfrA12*, *dfrA17*, *aadA2* and *aadA5* also detected within integrons.
(Nilima Priyadarshini Marhual, 2012)	*P. monodon*	Diseased farmed prawns	India	*V. alginolyticus* and *V. parahaemolyticus* (8)	Disc diffusion	**MAC‡, AGLY, PEN, CEPH-1/2, SUL,** CYPOL	
(Noor, Hasan and Rahman, 2014)	*M. rosenbergi* and *P. monodon*	Frozen retail shrimp	Bangladesh	*E. coli*, *Klebsiella*, *Shigella*, *Vibrio*, *Listeria and Staphylococci* (29)	Disc diffusion†	**CEPH-3/4/5‡, GLYP‡, MAC‡, QUIN‡, POLY‡, AGLY, PEN, AMPH, SUL, TET**	

(Otta, Karunasagar and Karunasagar, 2001)	*P. monodon*	Water from hatchery tanks	India	*Vibrio spp.* (87)	Disc diffusion	**POLY‡, PEN, CEPH-1/2, SUL, APEN**	
(Pan *et al.*, 2013)	*P. japonicus*, *P. chinensis* and *M. rosenbergii*	Retail shrimp	China	*V. vulnificus* (33)	Disc diffusion†	**AGLY, MONO, CEPH-1/2**	
(Rebouças *et al.*, 2011)	*L. vannamei*	Shrimp pond water, hatchery water, shrimp hepatopancreas	Brazil	*Vibrio spp.* (31)	Disc diffusion and broth macrodilution†	**CEPH-3/4/5‡, QUIN‡, SUL, TET**	Many resistance phenotypes were lost following plasmid curing (demonstrating that these genes were present on plasmids).
(Rocha, Sousa and Vieira, 2016)	*L. vannamei*	Farmed shrimp environment (estuary water and sediment)	Brazil	*Vibrio spp.* (70)	Disc diffusion†	**MONO, PEN, CEPH-1/2, AMPH, TET, NIT**	
(Roque *et al.*, 2001)	*Peneaus spp.*	Diseased and healthy shrimp, different tissues	Mexico	*Vibrio spp.* (144)	Disc diffusion	**QUIN‡, AMPH, SUL, TET**	
(Rortana *et al.*, 2018)	*L. vannamei*	Farmed shrimp and pond water	Thailand	*V. parahaemolyticus* (66)	Disc diffusion†	**MAC‡, PEN, TET, AMPH, SUL**	PCR detection of *qnrVC* and *pirAB-like* genes.
(S. Zhao *et al.*, 2018)	Does not state species	Farm pond water and sediment, water and larvae from hatcheries	China	*V. parahaemolyticus* (114)	Agar dilution†	**AGLY, PEN**	PCR detection of *qnrVC*, *blaCARB-17*, *blaTEM*, *floR*, *cat2*, *strA*, *strB*, *aac(3)-IV*, *tet(B)*, *tet(M)*, *arr*, *sul1*, and *sul2* genes.
(Shakir *et al.*, 2012)	*P. monodon*	Retail frozen shrimp	Thailand	*Aeromonas spp.* (317)	Broth microdilution†	**QUIN‡**	PCR detection of mutations in *qnrAB*, *gyrA*, *gyrB* and *parC*.
(Stalin and Srinivasan, 2016)	*P. monodon*	Shrimp pond sediment	India	*V. harveyi* (45)	Disc diffusion†	**GLYP‡, MAC‡, QUIN‡, AGLY, ANS, PEN, AMPH**	
(Su *et al.*, 2017)	*L. vannamei*	Shrimp gut, pond water, sediment, inflow water	China	All prokaryotes	16S amplicon sequencing and real-time	**QUIN‡, SUL, AMPH**	*sul1*, *qnrD*, *cmlA*, and *floR* were the predominant ARGs detected.

					qPCR to assess 14 AMR genes		
(Tendencia and Dela Peña, 2002)	*P. monodon*	Shrimp, pond water, sediment and farm soil	Philippines	*Vibrio spp* & other Gram negative organisms (including *Aeromonas spp.*, *Pseudomonas spp.*) (413)	Disc diffusion	**QUIN‡, TET**	
(Tran *et al.*, 2011)	*Penaeus spp.*	Imported frozen shrimp (into US)	India	*P. putida* (14 quinolone-resistant isolates)	Sensititre automated antimicrobial susceptibility system†	**CEPH-3/4/5‡, QUIN‡, AGLY, PEN, CEPH-1/2, SUL**	*qnrA* and *qnrB* genes detected on plasmids. Also novel substitutions in *gyrA*, *gyrB* and *parC*.
(Wong *et al.*, 2012)	Does not state species	Retail shrimp	Hong Kong	*V. parahemolyticus* (208)	Broth microdilution†	**CEPH-3/4/5‡, QUIN‡, AGLY, PEN, TET, AMPH**	IncN compatibility group plasmid detected, which carries *blaPER-1*.
(Y. Zhao *et al.*, 2018)	Freshwater shrimp	Shrimp gut, pond water and sediment	China	All prokaryotes	Metagenomic shotgun sequencing	Did not confirm phenotypic resistance.	*bacA* (conferring bacitran resistance - a **CYPOL**), *mexB* (conferring multidrug resistance to a number of antibiotics including **AGLY**, **QUIN***, **TET** and b-lactams such as **PEN** and **CEPH**s) and mexF genes detected (conferring multidrug resistance to antibiotics such as **QUIN*** and **AMPH**).
(Yano *et al.*, 2011)	*L. vannamei* and *P. monodon*	Farmed shrimp and commensal organisms	Thailand	Heterotrophs, including *L. garvieae*, *Aeromonas spp.*, *Vibrio spp.* (176 oxytetracycline-resistant isolates)	Broth microdilution†	**TET**	Resistance conferred by *tet(A)*, *tet(C)*, *tet(D)*, *tet(E)*, *tet(M)* and *tet(S)* genes.

(Yano *et al.*, 2014)	*L. vannamei* and *P. monodon*	Farmed shrimp and commensal organisms	Thailand	*V. parahaemolyticus*, *V vulnificus* and *V. cholerae* (235)	Broth microdilution†	**QUIN‡, PEN, TET**	PCR detection of *CARB-7*, *tetA*, *tetB* and *tetH*. *TetA* and *tetH* were lost after plasmid curing, suggesting their presence on plasmids.
(Yano *et al.*, 2015)	*P. monodon* and *L. vannamei*	Farmed shrimp and commensal organisms	Thailand	*Aeromonas spp.* (87)	Disc diffusion†	**CEPH-3/4/5‡, MAC‡, QUIN‡, CARB, PEN, CEPH-1/2, TET, SUL LIN**	
(Zhang, Li and Sun, 2011)	*L. vannamei*	Farm pond water and sediment, water and larvae from hatcheries	China	Heterotrophs (217)	Disc diffusion†	**MAC‡, QUIN‡, POLY‡, AGLY, PEN, CEPH-1/2, AMPH, SUL, TET**	

Supplementary Table 2 References

Albuquerque Costa, R. *et al.* (2015) 'Antibiotic-resistant vibrios in farmed shrimp', *BioMed Research International*, 2015. doi: 10.1155/2015/505914.

de Almeida, M. V. A. *et al.* (2017) 'Drug resistance, AmpC-β-lactamase and extended-spectrum β-lactamase-producing Enterobacteriaceae isolated from fish and shrimp', *Revista do Instituto de Medicina Tropical de Sao Paulo*. doi: 10.1590/S1678-9946201759070.

Arfatahery, N., Davoodabadi, A. and Abedimohtasab, T. (2016) 'Characterization of Toxin Genes and Antimicrobial Susceptibility of Staphylococcus aureus Isolates in Fishery Products in Iran', *Scientific Reports*. Nature Publishing Group, 6(October), pp. 1–7. doi: 10.1038/srep34216.

Banerjee, S. *et al.* (2012) 'Antibiotic resistant salmonella and Vibrio associated with farmed Litopenaeus vannamei', *The Scientific World Journal*, 2012. doi: 10.1100/2012/130136.

Dib, A. L. *et al.* (2018) 'Isolation, molecular characterization and antimicrobial resistance of enterobacteriaceae isolated from fish and seafood', *Food Control*. Elsevier Ltd, 88, pp. 54–60. doi: 10.1016/j.foodcont.2018.01.005.

Han, J. E. *et al.* (2015) 'Plasmid mediated tetracycline resistance of Vibrio parahaemolyticus associated with acute hepatopancreatic necrosis disease (AHPND) in shrimps', *Aquaculture Reports*. Elsevier B.V., 2, pp. 17–21. doi: 10.1016/j.aqrep.2015.04.003.

He, Y. *et al.* (2015) 'Detection and characterization of integrative and conjugative elements (ICEs)-positive Vibrio cholerae isolates from aquacultured shrimp and the environment in Shanghai, China', *Marine Pollution Bulletin*, 101(2), pp. 526–532. doi: 10.1016/j.marpolbul.2015.10.062.

He, Y. *et al.* (2016) 'Antibiotic and heavy-metal resistance of Vibrio parahaemolyticus isolated from fresh shrimps in Shanghai fish markets, China', *Environmental Science and Pollution Research*, 23(15), pp. 15033–15040. doi: 10.1007/s11356-016-6614-4.

Hua, L. M. and Apun, K. (2013) 'Antimicrobial Susceptibilities of Vibrio parahaemolyticus Isolates from Tiger Shrimps (Penaeus monodon) Aquaculture in Kuching, Sarawak', *Research Journal of Microbiology*, 8(1), pp. 55–62. doi: 10.3923/jm.2013.55.62.

Jana, T. K. *et al.* (2014) 'Management induced changes of antibiotic resistant strains of heterotrophic bacteria in shrimp farming ponds', *Indian Journal of Animal Sciences*.

Karunasagar, I. *et al.* (1994) 'Mass mortality of Penaeus monodon larvae due to antibiotic-resistant Vibrio harveyi infection', *Aquaculture*, 128(3–4), pp. 203–209. doi: 10.1016/0044-8486(94)90309-3.

Le, T. X., Munekage, Y. and Kato, S. I. (2005) 'Antibiotic resistance in bacteria from shrimp farming in mangrove areas', *Science of the Total Environment*, 349(1–3), pp. 95–105. doi: 10.1016/j.scitotenv.2005.01.006.

Letchumanan, V. *et al.* (2015) 'Occurrence and antibiotic resistance of Vibrio parahaemolyticus from Shellfish in Selangor, Malaysia', *Frontiers in Microbiology*, 6(DEC), pp. 1–11. doi: 10.3389/fmicb.2015.01417.

de Macedo, A. J. R. *et al.* (2016) 'Drug-resistant bacteria in frozen and fresh marine shrimp', *African Journal of Microbiology Research*, 10(33), pp. 1358–1362. doi: 10.5897/AJMR2016.7969.

de Melo, L. M. R. *et al.* (2011) 'Antibiotic resistance of Vibrio parahaemolyticus isolated from pond-reared Litopenaeus vannamei marketed in Natal, Brazil', *Brazilian Journal of Microbiology*. doi: 10.1590/S1517-83822011000400032.

Molina-Aja, A. *et al.* (2002) 'Plasmid profiling and antibiotic resistance of Vibrio strains isolated from cultured penaeid shrimp', *FEMS Microbiology Letters*. doi: 10.1016/S0378-1097(02)00791-7.

Nawaz, M. *et al.* (2012) 'Isolation and characterization of multidrug-resistant Klebsiella spp. isolated

from shrimp imported from Thailand', *International Journal of Food Microbiology*. Elsevier B.V., 155(3), pp. 179–184. doi: 10.1016/j.ijfoodmicro.2012.02.002.

Nawaz, M. *et al.* (2015) 'Characterisation of novel mutations involved in quinolone resistance in Escherichia coli isolated from imported shrimp', *International Journal of Antimicrobial Agents*. Elsevier B.V., 45(5), pp. 471–476. doi: 10.1016/j.ijantimicag.2014.11.010.

Nilima Priyadarshini Marhual (2012) 'Characterization of Vibrio alginolyticus and Vibrio parahaemolyticus isolated from Penaeus monodon: Antimicrobial resistance, plasmid profiles and random amplification of polymorphic DNA analysis', *African Journal of Microbiology Research*. doi: 10.5897/AJMR11.731.

Noor, R., Hasan, M. F. and Rahman, M. M. (2014) 'Molecular characterization of the virulent microorganisms along with their drug-resistance traits associated with the export quality frozen shrimps in Bangladesh', *SpringerPlus*, 3(1), pp. 1–7. doi: 10.1186/2193-1801-3-469.

Otta, Karunasagar and Karunasagar (2001) 'Bacteriological study of shrimp, penaeus monodon fabricius, hatcheries in India', *Journal of Applied Ichthyology*, 17(2), pp. 59–63. doi: 10.1046/j.1439-0426.2001.00249.x.

Pan, J. *et al.* (2013) 'Molecular characterization and antibiotic susceptibility of Vibrio vulnificus in retail shrimps in Hangzhou, People's Republic of China.', *J Food Prot*. doi: 10.4315/0362-028X.JFP-13-161.

Rebouças, R. H. *et al.* (2011) 'Antimicrobial resistance profile of Vibrio species isolated from marine shrimp farming environments (Litopenaeus vannamei) at Ceará, Brazil', *Environmental Research*, 111(1), pp. 21–24. doi: 10.1016/j.envres.2010.09.012.

Rocha, R. dos S., Sousa, O. V. de and Vieira, R. H. S. dos F. (2016) 'Multidrug-resistant Vibrio associated with an estuary affected by shrimp farming in Northeastern Brazil', *Marine Pollution Bulletin*. Elsevier Ltd, 105(1), pp. 337–340. doi: 10.1016/j.marpolbul.2016.02.001.

Roque, A. *et al.* (2001) 'In vitro susceptibility to 15 antibiotics of vibrios isolated from penaeid shrimps in Northwestern Mexico', *International Journal of Antimicrobial Agents*. doi: 10.1016/S0924-8579(01)00308-9.

Rortana, C. *et al.* (2018) 'Antimicrobial resistance and pirAB-like profiles of Vibrio parahaemolyticus in Pacific white shrimp', *Agriculture and Natural Resources*, 52, pp. 377–381. doi: 10.1016/j.anres.2018.10.010.

Shakir, Z. *et al.* (2012) 'Molecular characterization of fluoroquinolone-resistant aeromonas spp. isolated from imported shrimp', *Applied and Environmental Microbiology*, 78(22), pp. 8137–8141. doi: 10.1128/AEM.02081-12.

De Silva, B. C. J. *et al.* (2018) 'Frozen White-Leg Shrimp (*Litopenaeus vannamei*) in Korean Markets as a Source of *Aeromonas* spp. Harboring Antibiotic and Heavy Metal Resistance Genes', *Microbial Drug Resistance*, (June), p. mdr.2018.0035. doi: 10.1089/mdr.2018.0035.

Stalin, N. and Srinivasan, P. (2016) 'Molecular characterization of antibiotic resistant Vibrio harveyi isolated from shrimp aquaculture environment in the south east coast of India', *Microbial Pathogenesis*. Elsevier Ltd, 97, pp. 110–118. doi: 10.1016/j.micpath.2016.05.021.

Su, H. *et al.* (2017) 'Occurrence and temporal variation of antibiotic resistance genes (ARGs) in shrimp aquaculture: ARGs dissemination from farming source to reared organisms', *Science of the Total Environment*, 607–608, pp. 357–366. doi: 10.1016/j.scitotenv.2017.07.040.

Tendencia, E. A. and Dela Peña, L. D. (2002) 'Level and percentage recovery of resistance to oxytetracycline and oxolinic acid of bacteria from shrimp ponds', *Aquaculture*, 213(1–4), pp. 1–13. doi: 10.1016/S0044-8486(02)00017-0.

Tran, Q. T. *et al.* (2011) ‘Plasmid-mediated quinolone resistance in Pseudomonas putida isolates from imported shrimp’, *Applied and Environmental Microbiology*, 77(5), pp. 1885–1887. doi: 10.1128/AEM.01176-10.

Wong, M. H. Y. *et al.* (2012) ‘Characterization of extended-spectrum-β-lactamase-producing Vibrio parahaemolyticus’, *Antimicrobial Agents and Chemotherapy*, 56(7), pp. 4026–4028. doi: 10.1128/AAC.00385-12.

Yano, Y. *et al.* (2011) ‘Diversity and characterization of oxytetracycline-resistant bacteria associated with non-native species, white-leg shrimp (Litopenaeus vannamei), and native species, black tiger shrimp (Penaeus monodon), intensively cultured in Thailand’, *Journal of Applied Microbiology*. doi: 10.1111/j.1365-2672.2010.04926.x.

Yano, Y. *et al.* (2014) ‘Prevalence and antimicrobial susceptibility of Vibrio species related to food safety isolated from shrimp cultured at inland ponds in Thailand’, *Food Control*. Elsevier Ltd, 38(1), pp. 30–36. doi: 10.1016/j.foodcont.2013.09.019.

Yano, Y. *et al.* (2015) ‘Occurrence, molecular characterization, and antimicrobial susceptibility of Aeromonas spp. in marine species of shrimps cultured at inland low salinity ponds’, *Food Microbiology*. Elsevier Ltd, 47, pp. 21–27. doi: 10.1016/j.fm.2014.11.003.

Zhang, Y. Bin, Li, Y. and Sun, X. L. (2011) ‘Antibiotic resistance of bacteria isolated from shrimp hatcheries and cultural ponds on Donghai Island, China’, *Marine Pollution Bulletin*. Elsevier Ltd, 62(11), pp. 2299–2307. doi: 10.1016/j.marpolbul.2011.08.048.

Zhao, S. *et al.* (2018) ‘Antimicrobial resistance and pulsed-field gel electrophoresis typing of Vibrio parahaemolyticus isolated from shrimp mariculture environment along the east coast of China’, *Marine Pollution Bulletin*. Elsevier, 136(August), pp. 164–170. doi: 10.1016/j.marpolbul.2018.09.017.

Zhao, Y. *et al.* (2018) ‘Metagenomic analysis revealed the prevalence of antibiotic resistance genes in the gut and living environment of freshwater shrimp’, *Journal of Hazardous Materials*. Elsevier, 350(September 2017), pp. 10–18. doi: 10.1016/j.jhazmat.2018.02.004.
